# Association of Preterm Births Among US Latina Women With the 2016 Presidential Election

**DOI:** 10.1001/jamanetworkopen.2019.7084

**Published:** 2019-07-19

**Authors:** Alison Gemmill, Ralph Catalano, Joan A. Casey, Deborah Karasek, Héctor E. Alcalá, Holly Elser, Jacqueline M. Torres

**Affiliations:** 1 Program in Public Health, Department of Family, Population and Prevention Medicine, Stony Brook University, Stony Brook, New York; 2 currently affiliated with Department of Population, Family and Reproductive Health, Johns Hopkins Bloomberg School of Public Health, Baltimore, Maryland; 3 School of Public Health, University of California, Berkeley; 4 Preterm Birth Initiative, University of California, San Francisco; 5 Department of Epidemiology and Biostatistics, University of California, San Francisco

## Abstract

**Importance:**

The circumstances surrounding the 2016 US presidential election have been proposed as a significant stressor in the lives of the US Latino population. Few studies to date, however, have evaluated the population health implications of the election for Latina mothers and their children.

**Objective:**

To determine whether preterm births (gestational age, <37 weeks) among US Latina women increased above expected levels after the 2016 US presidential election.

**Design, Setting, and Participants:**

In this national population-based study, an interrupted time series design, used to evaluate whether policies or other population-level changes interrupt a trend in an outcome, compared monthly counts of preterm births to Latina women after the 2016 presidential election with the number expected had the election not taken place. Women residing in the United States who had singleton births during the study period were included. Counts of singleton term and preterm births by month and race/ethnicity from January 1, 2009, through July 30, 2017 (32 860 727 live births), were obtained from the Centers for Disease Control and Prevention Wonder online database. These methods were applied separately to male and female births. Data were analyzed from November 8, 2018, through May 7, 2019.

**Exposures:**

Pregnancy in the 2016 US presidential election.

**Main Outcomes and Measures:**

The number of male and female preterm births based on the last menstrual period.

**Results:**

Among the 32 860 727 live births recorded during the study period, 11.0% of male and 9.6% of female births to Latina women were preterm compared with 10.2% and 9.3%, respectively, to other women. In the 9-month period beginning with November 2016, an additional 1342 male (95% CI, 795-1889) and 995 female (95% CI, 554-1436) preterm births to Latina women were found above the expected number of preterm births had the election not occurred.

**Conclusions and Relevance:**

The 2016 US presidential election appears to have been associated with an increase in preterm births among US Latina women. Anti-immigration policies have been proposed and enforced in the aftermath of the 2016 presidential election; future research should evaluate the association of these actions with population health.

## Key Points

**Question:**

Did preterm births increase among Latina women who were pregnant during the 2016 US presidential election?

**Findings:**

This population-based study used an interrupted time series design to assess 32.9 million live births and found that the number of preterm births among Latina women increased above expected levels after the election.

**Meaning:**

The 2016 presidential election may have been associated with adverse health outcomes of Latina women and their newborns.

## Teaser

This national population-based study uses a quasi-experimental design to assess the association between preterm births among US Latina mothers and the 2016 US presidential election.

## Introduction

Speculation grows that the circumstances surrounding the 2016 presidential election may have had a uniquely negative effect on the health of the US Latino population.^1,2,3,4^ The campaign leading to the election was marked by highly racialized rhetoric and promises of punitive, anti-immigrant policies.^5^ Consequently, the 2016 election may have acutely stressed Latino immigrants and their US-born coethnic family members and communities and contributed to heightened fear of deportation and the potential reversal of proimmigrant legislation (eg, the Deferred Action for Childhood Arrivals program).^6,7,8,9^ Indeed, in the aftermath of the 2016 presidential election, nearly half of US-born Latinos and two-thirds of Latino immigrants reported fearing that a family member or close friend might be deported, regardless of their own status.^6,7^

Researchers have used birth outcomes as tracers of acute stress in a population. Preterm birth, in particular, appears to have distinct etiological linkages with maternal psychosocial stress. Although the biological mechanisms underlying this association remain unclear,^10,11^ myriad studies suggest that acute stressors may contribute to elevated risk for preterm birth through pathways of elevated systemic inflammation, immune dysregulation, increases in maternal and fetal cortisol levels, and the placental production of corticotropin-releasing hormone.^11,12^

Although research on the health effects of anti-immigration rhetoric and policies remains sparse at this time, studies have shown associations between immigration stress (eg, fear of deportation, perceptions of anti-immigration policies) and poorer mental health^13,14^ as well as higher systolic blood pressure and pulse pressure among Latina adults,^15^ which are known risk factors for preterm birth. Birth outcomes may also be affected by changes in health-seeking behavior; a recent study documented increases in inadequate prenatal care among US nonnative Latina women coincident with anti-immigration rhetoric.^16^

Two recent studies^17,18^ investigated how anti-immigration legislation and policing affected births among Latina women. The first study^17^ found a 24% greater risk of low birth weight among children born to Latina mothers after a federal immigration raid compared with births the year before the raid; no such change appeared among births to non-Latina women. The second study^18^ found that prenatal exposure to the passage of a restrictive immigration law in Arizona coincided with lower birth weight among children born to Latina immigrant women but not among children born to US-born white, black, or Latina women.

In the only study of the potential effect of the 2016 presidential election on birth outcomes, Krieger and colleagues^19^ found that the rate of preterm births among Latina women in New York, New York, increased from 7.7% before the inauguration to 8.2% after. Although Krieger et al^19^ provide evidence consistent with an association between the election and preterm births among Latina women, the methods the authors used did not adjust for secular trends, cycles, or other forms of temporal patterning that could lead to spurious findings. Because preterm birth varies seasonally,^20^ for example, a comparison between the periods before and after an event such as a presidential election should ensure that any association does not arise solely from seasonally expected shifts from lower to higher numbers of preterm births. Second, it remains unclear whether the patterns found in New York City generalize nationwide. Given that New York City has signaled support for immigrants by limiting cooperation between local agencies and federal immigration authorities,^21,22,23^ national data may show sharper increases in preterm births after the election.

We used national data and methods that control for temporal patterning to test the hypothesis that preterm births rose above otherwise expected levels among Latina women in the US after the 2016 election. We also tested our hypothesis separately for male and female births because research suggests that preterm birth and its sequelae appear to differ by sex of the fetus, with male infants appearing to be at elevated risk.^24^

## Methods

### Data and Measures

All analyses and reporting of results were conducted in accordance with the Strengthening the Reporting of Observational Studies in Epidemiology (STROBE) reporting guidelines for cohort studies.^25^ Institutional review board approval and informed consent were not required because the deidentified data are publicly available through a data use agreement with the National Center for Health Statistics.^26^

Our data came from the Centers for Disease Control and Prevention Wonder online database, which provides counts of live births in the United States by birth characteristics.^27^ Our dependent variables included monthly counts of male and female live births before 37 weeks’ gestation (ie, preterm) to mothers who self-identified as Hispanic (Latina) on the birth certificate. Maternal race/ethnicity was classified in accordance with the 1997 Office of Management and Budget standards.^28^ Covariates included monthly counts of male and female preterm births to non-Latina women as well as term births to Latina women. We defined gestational age based on the date of the last menstrual period to ensure consistency across time. As described below, we used 94 months of the presidency of Barack Obama (ie, January 21, 2009, through October 31, 2016) to estimate counterfactual values of preterm births to Latina women during the 9 months beginning November 1, 2016, and ending July 31, 2017.

### Statistical Analysis

Data were analyzed from November 8, 2018, through May 7, 2019. We tested our hypothesis with an approach commonly used to determine whether an acute environmental stressor coincides with changes in the characteristics of an exposed population.^29^ This interrupted time series approach compares values observed after the stressor has occurred with counterfactuals extrapolated from patterns in the prestressor data. These prestressor patterns presumably reflect the population’s adaptation to an environment possibly interrupted by the stressor. Our theory assumes that the policy and regulatory environment of the Obama administration constituted, in part, the environment to which Latina women, among others, had adapted for nearly 8 years and that Trump promised to change if elected. That is, we argue that the policy and regulatory environment promised under President Trump would be perceived as more hostile to Latina women when compared with the policy and regulatory environment they experienced under President Obama.

Our interrupted time series test proceeded through 4 steps. A detailed description of the statistical analysis, including test equations, can be found in eMethods 1 in the Supplement. First, we regressed separately the monthly number of preterm male and female births to Latina women for the 94 months before the 2016 election on the following 4 covariates: the monthly number of term births to Latina women in the same month as preterm births (ie, month *t*) as well as in the 2 months after (ie, month *t *+ 1 and month *t *+ 2) and the monthly number of preterm births to non-Latina women at month *t*. Including term births to Latina women in the model controls for the size of the population at risk; we specified these term births in months *t*, *t *+ 1, and *t *+ 2 because the conception cohort at risk of yielding preterm births in month *t* was likely born during those 3 months. Consistent with the comparison population design,^30,31^ we included preterm births to non-Latina women to control for patterns—seasonality, for example—that appear in the incidence of preterm birth regardless of race/ethnicity of the mother. Including preterm births to non-Latina women also helped control for unpatterned phenomena—such as changes in clinical practices or record-keeping procedures—that could affect temporal variation in all preterm births.

Second, we used the Box-Jenkins methods described by Box et al^32^ to detect autocorrelation, including trends, cycles (eg, seasonality), and/or the tendency to remain temporarily elevated or depressed after high or low values, in the residuals of the sex-specific models estimated in step 1. This autocorrelation would be unique to preterm births among Latina women because any such patterns shared with term births to Latina women or preterm births to women regardless of their race/ethnicity would be controlled in step 1. We converted the models estimated in step 1 to Box-Jenkins transfer functions that included coefficients specifying autocorrelation detected in step 2.^32^ Adding these coefficients not only ensured that our tests complied with the statistically important assumption of error terms free of autocorrelation but also precluded our finding a spurious association arising from a coincidence between the election and seasonally expected high counts of preterm births among Latina women.^30^

In step 3, we applied the transfer functions devised in step 2 (ie, those estimated for the 94 months of the Obama era) to 103 months ending July 2017 to estimate counterfactuals for the 9 birth cohorts in gestation at the election (ie, those born from November 2016 through July 2017). The argument that the 2016 election increased preterm birth among Latinas implies that the mean of the last 9 residuals of this model (ie, the observed less the counterfactual values for months 95 through 103) will significantly exceed the mean of all 103 residuals. In step 4, we determined whether the mean of the last 9 residuals of the model estimated in step 3 significantly (ie, *P* < .05; single-tailed test) exceeded the mean of all the residuals by regressing the 103 residuals on an exposure variable scored 1 for November through July 2016 and 0 otherwise.

Although we believe this test provides rigor and transparency, other perhaps less intuitive approaches could also apply. We pursued 2 of these to estimate the robustness of the results of our primary test. First, we proceeded through steps 1 and 2 above but used all 103 test cohorts. We then expanded the transfer function estimated in step 2 to include the binary election variable, thereby creating a model that simultaneously estimated coefficients for all the variables described above.^32^ We would infer support for our hypothesis if the coefficient for the election variable significantly exceeded 0.

In a second robustness check, we again implemented the first 2 steps described above for all 103 cohorts but then used the methods of Chang et al^33^ to detect segments of the residuals that formed not only level shifts such as what we hypothesized but also changes in slope and spike-and-decay sequences. Our theory implies a level shift at or near the election.

We also explored our data for other associations concerned with the timing of parturition. First, we analyzed birth cohort–specific associations with the election to detect plausible critical periods in pregnancy.^11^ We used analyses such as those described above, and in more detail in eMethods 1 in the Supplement, to determine which of the 9 birth cohorts in gestation at the time of the election exhibited the greatest response. Second, we applied outlier detection methods^33^ to the model estimated in step 4 to determine whether cohorts born before the election, but whose mothers were exposed to the rhetoric of the 2016 campaign (ie, first 10 months of 2016), may have yielded preterm births different from expected.

Following convention,^33^ we defined an outlying cohort conservatively (ie, 2-sided *P* < .005). Coefficients for the main analysis were obtained from the regression equation specified in step 4, and 95% CIs were calculated as the estimated coefficient plus or minus the product of 1.96 and the estimate’s standard error. All analyses were conducted with Scientific Computing Associates software.^34^ Code and output are available in eMethods 2 in the Supplement.

## Results

**Figure 1.**
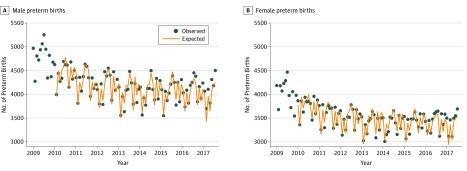
Observed and Expected Monthly Trend of Male and Female Preterm Births to Latina Women Includes 103 months ending July 2017. Expected values were generated from a time series model using data from 94 months of the presidency of Barack Obama (ie, January 2009 through October 2016). The first 13 months of the expected values for male births and first 12 months for female births were lost to modeling.

Our analyses included 16 825 845 live male and 16 034 882 live female singleton births (32 860 727 live births) from January 1, 2009, through July 30, 2017; nearly one-quarter of these births (23.5%) were to Latina women. Preterm infants represented 11.0% of male and 9.6% of female births to Latina women and 10.2% and 9.3% of those to other women. Figure 1 shows the expected monthly counts under the counterfactual scenario in which the 2016 election did not take place as well as the observed counts of male and female preterm births to Latina women during the test period. All birth count variables (ie, preterm births to Latina mothers, preterm births to other mothers, and term births to Latina mothers) exhibited strong seasonality for male and female births. Consistent with convention,^32^ we therefore differenced the birth count series at 12 months (ie, the number of births at month *t* subtracted from those at month 12) to remove seasonality.

The coefficient, estimated in step 4 above, for the exposure variable among male births was 149.1 (95% CI, 88.3-209.9), which implies that in the 9-month period beginning with November 2016, we observed 1342 male preterm births (ie, 149.1 × 9 months; 95% CI, 795-1889) above the 36 828 expected under the counterfactual scenario in which the 2016 presidential election had not occurred, with the expected number generated from the 94 months of preelection birth data. The exposure coefficient for female births was 110.6 (95% CI, 61.6-159.6), implying 995 more preterm births (95% CI, 554-1436) than the 30 867 that would have been expected based on preelection data. Together, we observed approximately 3.2% to 3.6% more preterm births to Latina women above expected levels of preterm births had the election not occurred.

**Figure 2.**
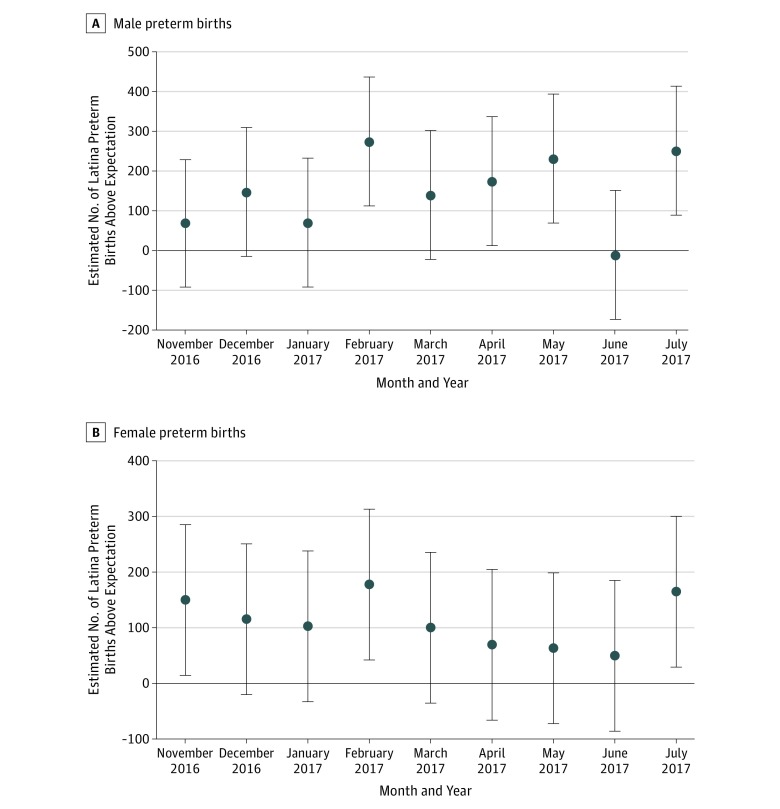
Monthly Coefficients for the Number of Male and Female Preterm Births to Latina Women Estimates are shown for the 9 birth cohorts in gestation during the presidential election of November 2016. Expected values were generated from a time series model using data from 94 months of the Obama presidency (ie, January 2009 through October 2016). Error bars indicate 95% CIs calculated as the estimate plus or minus the product of 1.96 and the estimate’s SE.

Results of testing for critical periods by gestational age at the time of the election found that preterm births peaked in February and July 2017 for male and female infants (Figure 2). Assuming, consistent with the existing literature, that the election rather than subsequent events marked the onset of stress among Latina women, these peaks would correspond to infants conceived (ie, born in July 2017) or in their second trimester of gestation (ie, born in February 2017) around the time of the election.

The results of our first robustness check in which we estimated a transfer function with all the cohorts and variables produced essentially the same results as our primary test. As described in more detail in eTables 1 to 3 in the Supplement, the election-variable coefficients for male and female births remained significantly greater than 0. The results of our second robustness check, in which we used the methods of Chang et al^33^ to detect level shifts, slope changes, and spike-and-decay sequences in the data, also converged with our primary tests. We found level shifts but no slope changes starting in August 2016 for male and October 2016 for female preterm births to Latina women.

## Discussion

In our analysis of all US births from 2009 to 2017, we found a significant upward level shift in the number of preterm births among US Latina women that coincided with the 2016 US presidential election. This result appeared most pronounced for infants conceived or in their second trimester of gestation near the time of the election. We found this evidence despite our conservative analytic approach, which controlled for potential concurrent but unrelated trends that might affect preterm birth. In other words, we observed an increase in Latina preterm births over and above levels expected from preterm birth in the general population. We also controlled for cycles and trends specific to preterm births among Latina women that could induce spurious associations in a simple, before-and-after study design.

Although the present study does not identify mechanisms underlying our findings, a growing body of evidence suggests that the circumstances surrounding the 2016 presidential election led to increased levels of psychosocial stress and anxiety among US immigrants and their coethnic family and community members.^6,7^ Moreover, prior research has suggested that uncertainty about the future of inclusive immigration policies and fear surrounding restrictive immigration enforcement are associated with poorer self-rated health,^8^ cardiometabolic risk factors,^15^ and inflammation,^35^ which may in turn contribute to increased risk for preterm birth.^12^ Changes in health behaviors, including accessing adequate prenatal care, may also be affected by immigration-related rhetoric, as suggested by a recent study among nearly 25 000 deliveries in Houston, Texas.^16^ Future research should investigate these potential mechanisms to uncover how the threat of punitive immigration laws and enforcement negatively affect population health outcomes, especially for pregnant women and their children.

Although our analyses do not differentiate between native and nonnative Latina women, we anticipate that had we been able to do so, the detected association would have been stronger among foreign-born Latina women.^18,19^ In data from New York City, Krieger et al^19^ found that an increase in preterm births to foreign-born Latina women was associated with observed increases among Latina women overall. Nevertheless, much research suggests potential spillover effects of anti-immigration rhetoric, policies, and policing on the broader Latino community, including members of mixed-status families (ie, families that include US-born individuals as well as immigrants who may be undocumented or hold other legal statuses).^3,17,35^ Most US Latino individuals, moreover, know someone who is undocumented, and one-third know someone who has experienced immigration detention or deportation.^36^ In addition, immigration enforcement relies on profiling of people who appear to be undocumented,^37^ thus placing Latino individuals, irrespective of documentation, at risk for profiling.

We also found evidence that the number of male and female preterm births over and above expected values peaked in February and July 2017. As noted above, these peaks would suggest critical periods near conception and during the second trimester, assuming the election marked the onset of stress. Other plausible stressors, however, followed near the election. The inauguration and subsequent passage of immigration-related Executive Orders in January 2017, for example, may have stressed Latina women as much as or more than the election. If so, the critical periods suggested by the February and July peaks would correspond to the late third trimester and middle first trimester, respectively. We know of no way to empirically discriminate between these competing inferences of critical periods.

### Limitations

Despite the strengths of this study, we acknowledge several limitations. First, we measured gestational age based on last menstrual period rather than the preferred measure based on obstetric estimate. We did this to ensure that gestational age is measured consistently during the study period, because the transition to the obstetric estimate standard in national data did not occur until 2014.^38^

Second, the publicly available data we used lacked information that would allow us to study other groups—persons of Middle Eastern and North African heritage, for example—targeted by anti-immigration rhetoric during the 2017 presidential inauguration.^1,2^ We focused on Latina women based on the available data, the compelling findings from Kreiger et al,^19^ and the growing body of evidence of anxiety in the Latino community due to the Trump presidency.^4,7^

Third, as noted above, we were not able to disaggregate births to Latina mothers by nativity status owing to data limitations. Foreign-born Latina women have a lower risk for preterm birth than their US-born counterparts.^39^ A decrease in the number of foreign-born women among Latina women giving birth immediately after the election could, therefore, have contributed to observed increases in preterm birth. If, however, compositional changes drove our results, we would expect a similar association between the election and male and female preterm births. Consistent with the literature reporting fetal sex differences in vulnerability to the maternal stress response,^40^ we found a greater response among male births.

Fourth, our hypothesis and study design only considered the 2016 election as a key environmental stressor. However, anti-immigration policies have been proposed and enforced repeatedly in the aftermath of the election, starting with the passage of 2017 immigration-related Executive Orders, the proposal to end the Deferred Action for Childhood Arrivals program, and the separation of immigrant families at the US-Mexico border, all of which may have contributed to ongoing stress that we did not capture in our study. Future research should continue to examine the effects of policy changes and their enforcement after the election.

## Conclusions

Given the rhetoric and policies promised under the Trump presidential campaign, the 2016 presidential election has been proposed as a significant stressor in the lives of US immigrants, their families, and their communities, with potentially uniquely acute effects on the US Latino population. We contribute to prior geographically focused research by evaluating the association of the 2016 presidential election with preterm births among Latina women using national data with an interrupted time series design that controlled for temporal variation that might otherwise lead to spurious findings. Our results suggest that the 2016 US presidential election was associated with an increase in preterm births among US Latina women.

## Notes

Supplement. **eMethods 1. **Data Analysis **eMethods 2. **Statistical Code and Output **eTable 1. **Estimated Parameters for Step 1 Regressions and Step 2 Transfer Functions for Monthly Male and Female Preterm Births to Latina Women in the United States for the First 94 Months of the Obama Presidency **eTable 2. **Estimated Parameters for Regressions of Male and Female Preterm Births to Latina Women on the Presidential Election of November 2016 **eTable 3.** Estimated Parameters for Full Transfer Functions for Monthly Male and Female Preterm Births to Latina Women in the United States for the 103 Months Beginning January 2009 **eReferences.**
