# Supplementary material for: Association of Preterm Births Among US Latina Women With the 2016 Presidential Election
Source: JAMA Netw Open. 2019 Jul 19;2(7):e197084. doi: 10.1001/jamanetworkopen.2019.7084 (PMC6647358; doi:10.1001/jamanetworkopen.2019.7084)
Supplement: Supplement. — eMethods 1. Data Analysis eMethods 2. Statistical Code and Output eTable 1. Estimated Parameters for Step 1 Regressions and Step 2 Transfer Functions for Monthly Male and Female Preterm Births to Latina Women in the United States for the First 94 Months of the Obama Presidency eTable 2. Estimated Parameters for Regressions of Male and Female Preterm Births to Latina Women on the Presidential Election of November 2016 eTable 3. Estimated Parameters for Full Transfer Functions for Monthly Male and Female Preterm Births to Latina Women in the United States for the 103 Months Beginning January 2009 eReferences. [file jamanetwopen-2-e197084-s001.pdf]

Supplementary Online Content

Gemmill A, Catalano R, Casey JA, et al. Association of preterm births among US Latina women with the 2016 presidential election. *JAMA Netw Open.* 2019;2(7):e197084. doi:10.1001/jamanetworkopen.2019.7084

eMethods 1. Data Analysis

eMethods 2. Statistical Code and Output

eTable 1. Estimated Parameters for Step 1 Regressions and Step 2 Transfer Functions for Monthly Male and Female Preterm Births to Latina Women in the United States for the First 94 Months of the Obama Presidency

eTable 2. Estimated Parameters for Regressions of Male and Female Preterm Births to Latina Women on the Presidential Election of November 2016

eTable 3. Estimated Parameters for Full Transfer Functions for Monthly Male and Female Preterm Births to Latina Women in the United States for the 103 Months Beginning January 2009

eReferences.

This supplementary material has been provided by the authors to give readers additional information about their work.

©2019 Gemmill A et al. *JAMA Network Open*

eMethods. Data Analysis

Step 1 We regressed the monthly number of preterm male and, separately, female births to Latina women for the 94 months before the 2016 election (i.e., the Obama environment) on the monthly number of *term* births to Latina women in the same month as preterm births (i.e., month *t*) as well as in the two months *after* (i.e., month *t*+1 and *t*+2), and on the monthly number of preterm births among non-Latina women at month *t*. All 3 birth count variables exhibited strong seasonality and were, consistent with convention,[1] differenced at 12 months (i.e., the number of births at month *t* subtracted from those at month *t*+12) to remove seasonality. We specified the regressions as follows.

$$\Delta^{12}Y_t = C + \beta_0\Delta^{12}X_{1t} + \beta_1\Delta^{12}X_{1t+1} + \beta_2\Delta^{12}X_{1t+2} + \beta_3\Delta^{12}X_{2t} + e_t$$

In which Y_t is the differenced monthly number of preterm male or female births to Latina women in the U.S. Δ^{12} is the "difference operator" that indicates Y, X_1, and X_2 at *t* were subtracted from Y, X_1, and X_2 at *t*-12 to remove seasonality from the series. C is a constant. β_0 through β_3 are estimated regression coefficients shown in **eTable 1** below. X_{1t} is the monthly number of term male or female births to Latina women in the U.S. X_{2t} is the monthly number of male or female preterm births to non-Latina women in the U.S. e is the error term at month t.

Step 2 We used Box and Jenkins methods[1] to detect autocorrelation including trends, cycles (e.g. seasonality), and/or the tendency to remain temporarily elevated or depressed after high or low values in the residuals of the sex-specific models estimated in step 1. We converted the models estimated in step 1 to Box-Jenkins transfer functions that included parameters that specify any autocorrelation detected in step 2. The general form of the transfer functions were as follows.

©2019 Gemmill A et al. *JAMA Network Open*

$$\Delta^{12}Y_t = C + \beta_0\Delta^{12}X_{1t} + \beta_1\Delta^{12}X_{1t+1} + \beta_2\Delta^{12}X_{1t+2} + \beta_3\Delta^{12}X_{2t} + (1-\theta B^q)/(1-\phi B^p)\alpha_t$$

In which Y, X_1, X_2, and Δ^{12} are as defined above. β_0 through β_3 are estimated coefficients shown in **eTable 1** below. ϕ is an autoregressive coefficient that expresses the tendency of Y to remain elevated or depressed after high or low values. B is the "backshift operator" or value of α at *t*-p or *t*-q. θ is a moving average coefficient that expresses the proportion of α at time *t*-1 carried into time *t*. α_t is the error term at month *t*.

Step 3 We applied the transfer functions devised in step 2 (i.e., those estimated for the 94 Obama era months) to 103 months ending July 2017 to estimate counterfactuals for the 9 birth cohorts in gestation at the election (i.e. those born from November 2016 through July 2017). We fixed the coefficients in the transfer functions to those estimated in step 2 for the 94 Obama era months. The counterfactuals and observed values appear in **Figure 1** of the main text. The argument that the Trump election increased preterm birth among Latinas implies that the mean of the last 9 residuals of this model (i.e., the observed values less the counterfactual values for months 95 through 103) will significantly exceed the mean of all 103 residuals.

Step 4 To determine if the mean of the last 9 residuals of the model estimated in step 3 differed significantly from the mean of all 103 residuals, we regressed the 103 residuals from the transfer function estimated in step 3 on an exposure variable scored 1 for November through July 2016 and 0 otherwise. The results, as described in the main text, were as follows.

Males: $\alpha_{mt} = -1.28 + 149.14X_t + e_t$

Females: $\alpha_{ft} = 0.19 + 110.58X_t + e_t$

©2019 Gemmill A et al. *JAMA Network Open*

Standard errors for the coefficients on X were 31.02 and 25.02 respectively for males and females. Standard errors for the constants were 9.8 for males and 7.92 for females. The error terms for both regressions had means of 0, constant variance, and exhibited no autocorrelation.

Critical Period Exploration As noted in the main text, we explored birth cohort-specific associations with the election to detect plausible "critical periods" in pregnancy. We regressed the 103 residuals estimated in step 3 on an "election indicator" variable scored 1 for November 2016 and 0 otherwise. The binary variable was specified in the synchronous configuration in which preterm births and the election indicator variable were in the same month, as well as in 8 configurations in which the election preceded births by 1 through 8 months. The results of this regression appear in **eTable 2** below and in **Figure 2** of the main text.

Alternative Tests We conducted two additional tests, as described in the main text, to determine how sensitive our results were to analytic approach. In the first, we proceeded through steps 1 and 2 above but used all 103 monthly test cohorts. We then expanded the transfer function estimated in step 2 to include the binary election variable. This model provided estimates of the unique contribution of all the independent variables described above to temporal variation in sex-specific preterm births to Latina women. **eTable 3** shows the results of this estimation. We infer support for our hypothesis given that the coefficients for the election variable significantly exceed 0 for males (177.80; standard error = 41.73) as well as females (112.77; standard error = 27.82).

In a second robustness check, we again implemented the first two steps described above for all 103 cohorts but then used the methods of Chang, Tiao, and Chen to detect segments of the residuals that formed not only level shifts such as those we hypothesized, but also changes in slope, as well as spike-and-decay sequences.[2] Given the exploratory nature of this approach, we set significance as segments likely to occur by chance once in 100 experiments. Our theory implies a level shift at or near the election. The Chang, Tiao, and Chen method detected an upward level shift among males born to Latina women that included the months August 2016 through July 2017. An upward level shift including October 2016 through July 2017 appeared for females. Other detected segments included 4 decaying spikes for males (down in May 2012 and March 2013; up in October 2012 and November 2013) and one decaying spike (down in March 2014) for females. No slope changes appeared for either sex.

©2019 Gemmill A et al. *JAMA Network Open*

eMethods 2. Statistical Code and Output

```
THE PC SCA STATISTICAL SYSTEM ( Release 5.2-Enterprise )

COPYRIGHT 1985-1998, SCIENTIFIC COMPUTING ASSOCIATES. ALL RIGHTS RESERVED
SCA PRODUCT  IDENTIFICATION: UTS & EXPERT (GSA OPTIONAL)
SCA SOFTWARE IDENTIFICATION: UC-BERKELEY.NETW ( 98110618 )
RELEASE  DATE:   9/ 1/97

SIZE OF WORKSPACE IS  999000  SINGLE PRECISION WORDS
DATE --  2/ 2/**              TIME -- 23:48:24
--



Input year,month,@
dsfall,nsfall,dsfhisp,nsfhisp,@
dsmall,nsmall,dsmhisp,nsmhisp

YEAR    , A  132  BY   1 VARIABLE, IS STORED IN THE WORKSPACE
MONTH   , A  132  BY   1 VARIABLE, IS STORED IN THE WORKSPACE
DSFALL  , A  132  BY   1 VARIABLE, IS STORED IN THE WORKSPACE
NSFALL  , A  132  BY   1 VARIABLE, IS STORED IN THE WORKSPACE
DSFHISP , A  132  BY   1 VARIABLE, IS STORED IN THE WORKSPACE
NSFHISP , A  132  BY   1 VARIABLE, IS STORED IN THE WORKSPACE
DSMALL  , A  132  BY   1 VARIABLE, IS STORED IN THE WORKSPACE
NSMALL  , A  132  BY   1 VARIABLE, IS STORED IN THE WORKSPACE
DSMHISP , A  132  BY   1 VARIABLE, IS STORED IN THE WORKSPACE
NSMHISP , A  132  BY   1 VARIABLE, IS STORED IN THE WORKSPACE
--


Pro out brief.

PROFILE OF THE CURRENT SESSION:

STYLE OF THIS SESSION . . .   PARTIAL
PROMPTING OF THIS SESSION .    NORMAL
ECHO OF THE INSTRUCTION . .       YES
LEVEL OF OUTPUT . . . . . .     BRIEF
COMPUTATION PRECISION . . .    SINGLE
FILE FOR INSTRUCTIONS . . .         6
FILE FOR OUTPUT . . . . . .        10
WIDTH OF INPUT DEVICE . . .        72
WIDTH OF OUTPUT DEVICE. . .        80
--


Workspace compressed. Novar 1000

THE WORKSPACE IS COMPRESSED
THE MAXIMUM NUMBER OF VARIABLES IS EXPANDED TO1018
--


Sel old year,month,dsfall,nsfall,dsfhisp,@
nsfhisp,dsmall,nsmall,dsmhisp,nsmhisp.@
new years,months,dsfalls,nsfalls,dsfhisps,@
nsfhisps,dsmalls,nsmalls,dsmhisps,nsmhisps.@
span (25,127)
```

```
VARIABLE   YEAR   IS EDITED, THE RESULT IS STORED IN VARIABLE  YEARS
VARIABLE  YEARS   IS  A  103  BY   1  MATRIX
VARIABLE  MONTH   IS EDITED, THE RESULT IS STORED IN VARIABLE  MONTHS
VARIABLE  MONTHS  IS  A  103  BY   1  MATRIX
VARIABLE  DSFALL  IS EDITED, THE RESULT IS STORED IN VARIABLE DSFALLS
VARIABLE DSFALLS  IS  A  103  BY   1  MATRIX
VARIABLE  NSFALL  IS EDITED, THE RESULT IS STORED IN VARIABLE NSFALLS
VARIABLE NSFALLS  IS  A  103  BY   1  MATRIX
VARIABLE DSFHISP  IS EDITED, THE RESULT IS STORED IN VARIABLE DSFHISPS
VARIABLE DSFHISPS IS  A  103  BY   1  MATRIX
VARIABLE NSFHISP  IS EDITED, THE RESULT IS STORED IN VARIABLE NSFHISPS
VARIABLE NSFHISPS IS  A  103  BY   1  MATRIX
VARIABLE  DSMALL  IS EDITED, THE RESULT IS STORED IN VARIABLE DSMALLS
VARIABLE DSMALLS  IS  A  103  BY   1  MATRIX
VARIABLE  NSMALL  IS EDITED, THE RESULT IS STORED IN VARIABLE NSMALLS
VARIABLE NSMALLS  IS  A  103  BY   1  MATRIX
VARIABLE DSMHISP  IS EDITED, THE RESULT IS STORED IN VARIABLE DSMHISPS
VARIABLE DSMHISPS IS  A  103  BY   1  MATRIX
VARIABLE NSMHISP  IS EDITED, THE RESULT IS STORED IN VARIABLE NSMHISPS
VARIABLE NSMHISPS IS  A  103  BY   1  MATRIX
--


nov16=years-years
--


Nov16(95)=1
--


n16step=nov16
--


n16step(96)=1
--


n16step(97)=1
--


n16step(98)=1
--


n16step(99)=1
--


n16step(100)=1
--


n16step(101)=1
--


n16step(102)=1
--
```

```
n16step(103)=1
--


prin years,months,nov16,n16step

YEARS    IS  A   103  BY     1  VARIABLE
MONTHS   IS  A   103  BY     1  VARIABLE
NOV16    IS  A   103  BY     1  VARIABLE
N16STEP  IS  A   103  BY     1  VARIABLE


VARIABLE      YEARS    MONTHS     NOV16   N16STEP
COLUMN-->       1         1         1         1
  ROW
    1     2009.000     1.000      .000      .000
    2     2009.000     2.000      .000      .000
    3     2009.000     3.000      .000      .000
    4     2009.000     4.000      .000      .000
    5     2009.000     5.000      .000      .000
    6     2009.000     6.000      .000      .000
    7     2009.000     7.000      .000      .000
    8     2009.000     8.000      .000      .000
    9     2009.000     9.000      .000      .000
   10     2009.000    10.000      .000      .000
   11     2009.000    11.000      .000      .000
   12     2009.000    12.000      .000      .000
   13     2010.000     1.000      .000      .000
   14     2010.000     2.000      .000      .000
   15     2010.000     3.000      .000      .000
   16     2010.000     4.000      .000      .000
   17     2010.000     5.000      .000      .000
   18     2010.000     6.000      .000      .000
   19     2010.000     7.000      .000      .000
   20     2010.000     8.000      .000      .000
   21     2010.000     9.000      .000      .000
   22     2010.000    10.000      .000      .000
   23     2010.000    11.000      .000      .000
   24     2010.000    12.000      .000      .000
   25     2011.000     1.000      .000      .000
   26     2011.000     2.000      .000      .000
   27     2011.000     3.000      .000      .000
   28     2011.000     4.000      .000      .000
   29     2011.000     5.000      .000      .000
   30     2011.000     6.000      .000      .000
   31     2011.000     7.000      .000      .000
   32     2011.000     8.000      .000      .000
   33     2011.000     9.000      .000      .000
   34     2011.000    10.000      .000      .000
   35     2011.000    11.000      .000      .000
   36     2011.000    12.000      .000      .000
   37     2012.000     1.000      .000      .000
   38     2012.000     2.000      .000      .000
   39     2012.000     3.000      .000      .000
   40     2012.000     4.000      .000      .000
   41     2012.000     5.000      .000      .000
   42     2012.000     6.000      .000      .000
   43     2012.000     7.000      .000      .000
   44     2012.000     8.000      .000      .000
   45     2012.000     9.000      .000      .000
   46     2012.000    10.000      .000      .000
   47     2012.000    11.000      .000      .000
   48     2012.000    12.000      .000      .000
```

49	2013.000	1.000	.000	.000
50	2013.000	2.000	.000	.000
51	2013.000	3.000	.000	.000
52	2013.000	4.000	.000	.000
53	2013.000	5.000	.000	.000
54	2013.000	6.000	.000	.000
55	2013.000	7.000	.000	.000
56	2013.000	8.000	.000	.000
57	2013.000	9.000	.000	.000
58	2013.000	10.000	.000	.000
59	2013.000	11.000	.000	.000
60	2013.000	12.000	.000	.000
61	2014.000	1.000	.000	.000
62	2014.000	2.000	.000	.000
63	2014.000	3.000	.000	.000
64	2014.000	4.000	.000	.000
65	2014.000	5.000	.000	.000
66	2014.000	6.000	.000	.000
67	2014.000	7.000	.000	.000
68	2014.000	8.000	.000	.000
69	2014.000	9.000	.000	.000
70	2014.000	10.000	.000	.000
71	2014.000	11.000	.000	.000
72	2014.000	12.000	.000	.000
73	2015.000	1.000	.000	.000
74	2015.000	2.000	.000	.000
75	2015.000	3.000	.000	.000
76	2015.000	4.000	.000	.000
77	2015.000	5.000	.000	.000
78	2015.000	6.000	.000	.000
79	2015.000	7.000	.000	.000
80	2015.000	8.000	.000	.000
81	2015.000	9.000	.000	.000
82	2015.000	10.000	.000	.000
83	2015.000	11.000	.000	.000
84	2015.000	12.000	.000	.000
85	2016.000	1.000	.000	.000
86	2016.000	2.000	.000	.000
87	2016.000	3.000	.000	.000
88	2016.000	4.000	.000	.000
89	2016.000	5.000	.000	.000
90	2016.000	6.000	.000	.000
91	2016.000	7.000	.000	.000
92	2016.000	8.000	.000	.000
93	2016.000	9.000	.000	.000
94	2016.000	10.000	.000	.000
95	2016.000	11.000	1.000	1.000
96	2016.000	12.000	.000	1.000
97	2017.000	1.000	.000	1.000
98	2017.000	2.000	.000	1.000
99	2017.000	3.000	.000	1.000
100	2017.000	4.000	.000	1.000
101	2017.000	5.000	.000	1.000
102	2017.000	6.000	.000	1.000
103	2017.000	7.000	.000	1.000

--

PSHis=nsmhisps+nsfhisps

--

Step1=nsfalls+nsmalls

```
--


PSNHIS=step1-pshis
--


TSHIS=dsfhisps+dsmhisps
--


TSHISa=dsfhisp+dsmhisp
--


Psmhis=nsmhisps
--


psmnh=nsmalls-psmhis
--


psfhis=nsfhisps
--


psfnh=nsfalls-psfhis
--


tsmhis=dsmhisps
--


tsmhisa=dsmhisp
--


tsfhis=dsfhisps
--


tsfhisa=dsfhisp
--


sel old tsmhisa,tsfhisa,tshisa.@
new tsmhis1,tsfhis1,tshis1. Span (26,128)

VARIABLE TSMHISA  IS EDITED, THE RESULT IS STORED IN VARIABLE TSMHIS1
VARIABLE TSMHIS1  IS  A  103  BY   1  MATRIX
VARIABLE TSFHISA  IS EDITED, THE RESULT IS STORED IN VARIABLE TSFHIS1
VARIABLE TSFHIS1  IS  A  103  BY   1  MATRIX
VARIABLE  TSHISA  IS EDITED, THE RESULT IS STORED IN VARIABLE  TSHIS1
VARIABLE  TSHIS1  IS  A  103  BY   1  MATRIX
--


sel old tsmhisa,tsfhisa,tshisa.@
new tsmhis2,tsfhis2,tshis2. Span (27,129)

VARIABLE TSMHISA  IS EDITED, THE RESULT IS STORED IN VARIABLE TSMHIS2
```

```
VARIABLE TSMHIS2  IS  A  103  BY   1  MATRIX
VARIABLE TSFHISA  IS EDITED, THE RESULT IS STORED IN VARIABLE TSFHIS2
VARIABLE TSFHIS2  IS  A  103  BY   1  MATRIX
VARIABLE  TSHISA  IS EDITED, THE RESULT IS STORED IN VARIABLE  TSHIS2
VARIABLE  TSHIS2  IS  A  103  BY   1  MATRIX
--


prin nov16,tshis,tshis1,tshis2

NOV16    IS  A   103  BY     1  VARIABLE
TSHIS    IS  A   103  BY     1  VARIABLE
TSHIS1   IS  A   103  BY     1  VARIABLE
TSHIS2   IS  A   103  BY     1  VARIABLE


VARIABLE     NOV16     TSHIS    TSHIS1    TSHIS2
COLUMN-->       1         1         1         1
  ROW
    1         .000 82043.000 74331.000 80369.000
    2         .000 74331.000 80369.000 77249.000
    3         .000 80369.000 77249.000 80046.000
    4         .000 77249.000 80046.000 81471.000
    5         .000 80046.000 81471.000 87418.000
    6         .000 81471.000 87418.000 86838.000
    7         .000 87418.000 86838.000 86514.000
    8         .000 86838.000 86514.000 82312.000
    9         .000 86514.000 82312.000 76246.000
   10         .000 82312.000 76246.000 79986.000
   11         .000 76246.000 79986.000 76611.000
   12         .000 79986.000 76611.000 69403.000
   13         .000 76611.000 69403.000 76418.000
   14         .000 69403.000 76418.000 72605.000
   15         .000 76418.000 72605.000 73894.000
   16         .000 72605.000 73894.000 75228.000
   17         .000 73894.000 75228.000 79666.000
   18         .000 75228.000 79666.000 81956.000
   19         .000 79666.000 81956.000 81897.000
   20         .000 81956.000 81897.000 78406.000
   21         .000 81897.000 78406.000 76150.000
   22         .000 78406.000 76150.000 79031.000
   23         .000 76150.000 79031.000 74609.000
   24         .000 79031.000 74609.000 67912.000
   25         .000 74609.000 67912.000 73713.000
   26         .000 67912.000 73713.000 69578.000
   27         .000 73713.000 69578.000 72602.000
   28         .000 69578.000 72602.000 75148.000
   29         .000 72602.000 75148.000 77988.000
   30         .000 75148.000 77988.000 81848.000
   31         .000 77988.000 81848.000 79186.000
   32         .000 81848.000 79186.000 74760.000
   33         .000 79186.000 74760.000 73196.000
   34         .000 74760.000 73196.000 74626.000
   35         .000 73196.000 74626.000 72585.000
   36         .000 74626.000 72585.000 67882.000
   37         .000 72585.000 67882.000 71769.000
   38         .000 67882.000 71769.000 66814.000
   39         .000 71769.000 66814.000 71690.000
   40         .000 66814.000 71690.000 72667.000
   41         .000 71690.000 72667.000 78536.000
   42         .000 72667.000 78536.000 81910.000
   43         .000 78536.000 81910.000 78027.000
   44         .000 81910.000 78027.000 77346.000
```

```
   45          .000 78027.000 77346.000 73010.000
   46          .000 77346.000 73010.000 73281.000
   47          .000 73010.000 73281.000 73172.000
   48          .000 73281.000 73172.000 64785.000
   49          .000 73172.000 64785.000 69786.000
   50          .000 64785.000 69786.000 67505.000
   51          .000 69786.000 67505.000 71762.000
   52          .000 67505.000 71762.000 70495.000
   53          .000 71762.000 70495.000 77879.000
   54          .000 70495.000 77879.000 79946.000
   55          .000 77879.000 79946.000 77637.000
   56          .000 79946.000 77637.000 76700.000
   57          .000 77637.000 76700.000 72359.000
   58          .000 76700.000 72359.000 75977.000
   59          .000 72359.000 75977.000 73886.000
   60          .000 75977.000 73886.000 65872.000
   61          .000 73886.000 65872.000 70755.000
   62          .000 65872.000 70755.000 68848.000
   63          .000 70755.000 68848.000 72477.000
   64          .000 68848.000 72477.000 71944.000
   65          .000 72477.000 71944.000 79304.000
   66          .000 71944.000 79304.000 80235.000
   67          .000 79304.000 80235.000 80138.000
   68          .000 80235.000 80138.000 77463.000
   69          .000 80138.000 77463.000 72564.000
   70          .000 77463.000 72564.000 77097.000
   71          .000 72564.000 77097.000 74037.000
   72          .000 77097.000 74037.000 67293.000
   73          .000 74037.000 67293.000 72885.000
   74          .000 67293.000 72885.000 70295.000
   75          .000 72885.000 70295.000 72265.000
   76          .000 70295.000 72265.000 73530.000
   77          .000 72265.000 73530.000 79474.000
   78          .000 73530.000 79474.000 81239.000
   79          .000 79474.000 81239.000 80742.000
   80          .000 81239.000 80742.000 77526.000
   81          .000 80742.000 77526.000 73336.000
   82          .000 77526.000 73336.000 77375.000
   83          .000 73336.000 77375.000 73079.000
   84          .000 77375.000 73079.000 69007.000
   85          .000 73079.000 69007.000 72682.000
   86          .000 69007.000 72682.000 69317.000
   87          .000 72682.000 69317.000 72337.000
   88          .000 69317.000 72337.000 73701.000
   89          .000 72337.000 73701.000 77243.000
   90          .000 73701.000 77243.000 82122.000
   91          .000 77243.000 82122.000 80039.000
   92          .000 82122.000 80039.000 76463.000
   93          .000 80039.000 76463.000 73388.000
   94          .000 76463.000 73388.000 75020.000
   95         1.000 73388.000 75020.000 72312.000
   96          .000 75020.000 72312.000 65317.000
   97          .000 72312.000 65317.000 70995.000
   98          .000 65317.000 70995.000 66348.000
   99          .000 70995.000 66348.000 71662.000
  100          .000 66348.000 71662.000 72366.000
  101          .000 71662.000 72366.000 76351.000
  102          .000 72366.000 76351.000 80438.000
  103          .000 76351.000 80438.000 77804.000
--


prin nov16,tsmhis,tsmhis1,tsmhis2
```

```
NOV16    IS  A   103  BY     1  VARIABLE
TSMHIS   IS  A   103  BY     1  VARIABLE
TSMHIS1  IS  A   103  BY     1  VARIABLE
TSMHIS2  IS  A   103  BY     1  VARIABLE
```

VARIABLE	NOV16	TSMHIS	TSMHIS1	TSMHIS2
COLUMN-->	1	1	1	1
ROW				
1	.000	41560.000	37834.000	41105.000
2	.000	37834.000	41105.000	39509.000
3	.000	41105.000	39509.000	41120.000
4	.000	39509.000	41120.000	41895.000
5	.000	41120.000	41895.000	44597.000
6	.000	41895.000	44597.000	44580.000
7	.000	44597.000	44580.000	44079.000
8	.000	44580.000	44079.000	42130.000
9	.000	44079.000	42130.000	39163.000
10	.000	42130.000	39163.000	40680.000
11	.000	39163.000	40680.000	38874.000
12	.000	40680.000	38874.000	35514.000
13	.000	38874.000	35514.000	38830.000
14	.000	35514.000	38830.000	37044.000
15	.000	38830.000	37044.000	37852.000
16	.000	37044.000	37852.000	38484.000
17	.000	37852.000	38484.000	40614.000
18	.000	38484.000	40614.000	41601.000
19	.000	40614.000	41601.000	41692.000
20	.000	41601.000	41692.000	39837.000
21	.000	41692.000	39837.000	39065.000
22	.000	39837.000	39065.000	40049.000
23	.000	39065.000	40049.000	37849.000
24	.000	40049.000	37849.000	34691.000
25	.000	37849.000	34691.000	37467.000
26	.000	34691.000	37467.000	35788.000
27	.000	37467.000	35788.000	37256.000
28	.000	35788.000	37256.000	38458.000
29	.000	37256.000	38458.000	40109.000
30	.000	38458.000	40109.000	41842.000
31	.000	40109.000	41842.000	40299.000
32	.000	41842.000	40299.000	37858.000
33	.000	40299.000	37858.000	37500.000
34	.000	37858.000	37500.000	37795.000
35	.000	37500.000	37795.000	36697.000
36	.000	37795.000	36697.000	34597.000
37	.000	36697.000	34597.000	36643.000
38	.000	34597.000	36643.000	34174.000
39	.000	36643.000	34174.000	36760.000
40	.000	34174.000	36760.000	37186.000
41	.000	36760.000	37186.000	40202.000
42	.000	37186.000	40202.000	41460.000
43	.000	40202.000	41460.000	39850.000
44	.000	41460.000	39850.000	39401.000
45	.000	39850.000	39401.000	36880.000
46	.000	39401.000	36880.000	37128.000
47	.000	36880.000	37128.000	37137.000
48	.000	37128.000	37137.000	32870.000
49	.000	37137.000	32870.000	35985.000
50	.000	32870.000	35985.000	34546.000
51	.000	35985.000	34546.000	36504.000
52	.000	34546.000	36504.000	36093.000
53	.000	36504.000	36093.000	39906.000

©2019 Gemmill A et al. *JAMA Network Open*

```
   54         .000 36093.000 39906.000 40721.000
   55         .000 39906.000 40721.000 39460.000
   56         .000 40721.000 39460.000 39161.000
   57         .000 39460.000 39161.000 37109.000
   58         .000 39161.000 37109.000 38931.000
   59         .000 37109.000 38931.000 37793.000
   60         .000 38931.000 37793.000 33760.000
   61         .000 37793.000 33760.000 36274.000
   62         .000 33760.000 36274.000 35264.000
   63         .000 36274.000 35264.000 37000.000
   64         .000 35264.000 37000.000 36705.000
   65         .000 37000.000 36705.000 40502.000
   66         .000 36705.000 40502.000 40978.000
   67         .000 40502.000 40978.000 40682.000
   68         .000 40978.000 40682.000 39361.000
   69         .000 40682.000 39361.000 36929.000
   70         .000 39361.000 36929.000 39433.000
   71         .000 36929.000 39433.000 37748.000
   72         .000 39433.000 37748.000 34274.000
   73         .000 37748.000 34274.000 37228.000
   74         .000 34274.000 37228.000 36278.000
   75         .000 37228.000 36278.000 36892.000
   76         .000 36278.000 36892.000 37649.000
   77         .000 36892.000 37649.000 40555.000
   78         .000 37649.000 40555.000 41457.000
   79         .000 40555.000 41457.000 41078.000
   80         .000 41457.000 41078.000 39688.000
   81         .000 41078.000 39688.000 37413.000
   82         .000 39688.000 37413.000 39447.000
   83         .000 37413.000 39447.000 37129.000
   84         .000 39447.000 37129.000 35190.000
   85         .000 37129.000 35190.000 37309.000
   86         .000 35190.000 37309.000 35490.000
   87         .000 37309.000 35490.000 36990.000
   88         .000 35490.000 36990.000 37761.000
   89         .000 36990.000 37761.000 39423.000
   90         .000 37761.000 39423.000 41710.000
   91         .000 39423.000 41710.000 40827.000
   92         .000 41710.000 40827.000 39088.000
   93         .000 40827.000 39088.000 37482.000
   94         .000 39088.000 37482.000 38212.000
   95        1.000 37482.000 38212.000 36840.000
   96         .000 38212.000 36840.000 33251.000
   97         .000 36840.000 33251.000 36168.000
   98         .000 33251.000 36168.000 33941.000
   99         .000 36168.000 33941.000 36626.000
  100         .000 33941.000 36626.000 37108.000
  101         .000 36626.000 37108.000 38857.000
  102         .000 37108.000 38857.000 41094.000
  103         .000 38857.000 41094.000 39556.000
--


prin nov16,tsfhis,tsfhis1,tsfhis2

NOV16    IS  A   103  BY     1  VARIABLE
TSFHIS   IS  A   103  BY     1  VARIABLE
TSFHIS1  IS  A   103  BY     1  VARIABLE
TSFHIS2  IS  A   103  BY     1  VARIABLE


VARIABLE      NOV16    TSFHIS   TSFHIS1   TSFHIS2
COLUMN-->        1          1         1         1
```

©2019 Gemmill A et al. *JAMA Network Open*

ROW				
1	.000	40483.000	36497.000	39264.000
2	.000	36497.000	39264.000	37740.000
3	.000	39264.000	37740.000	38926.000
4	.000	37740.000	38926.000	39576.000
5	.000	38926.000	39576.000	42821.000
6	.000	39576.000	42821.000	42258.000
7	.000	42821.000	42258.000	42435.000
8	.000	42258.000	42435.000	40182.000
9	.000	42435.000	40182.000	37083.000
10	.000	40182.000	37083.000	39306.000
11	.000	37083.000	39306.000	37737.000
12	.000	39306.000	37737.000	33889.000
13	.000	37737.000	33889.000	37588.000
14	.000	33889.000	37588.000	35561.000
15	.000	37588.000	35561.000	36042.000
16	.000	35561.000	36042.000	36744.000
17	.000	36042.000	36744.000	39052.000
18	.000	36744.000	39052.000	40355.000
19	.000	39052.000	40355.000	40205.000
20	.000	40355.000	40205.000	38569.000
21	.000	40205.000	38569.000	37085.000
22	.000	38569.000	37085.000	38982.000
23	.000	37085.000	38982.000	36760.000
24	.000	38982.000	36760.000	33221.000
25	.000	36760.000	33221.000	36246.000
26	.000	33221.000	36246.000	33790.000
27	.000	36246.000	33790.000	35346.000
28	.000	33790.000	35346.000	36690.000
29	.000	35346.000	36690.000	37879.000
30	.000	36690.000	37879.000	40006.000
31	.000	37879.000	40006.000	38887.000
32	.000	40006.000	38887.000	36902.000
33	.000	38887.000	36902.000	35696.000
34	.000	36902.000	35696.000	36831.000
35	.000	35696.000	36831.000	35888.000
36	.000	36831.000	35888.000	33285.000
37	.000	35888.000	33285.000	35126.000
38	.000	33285.000	35126.000	32640.000
39	.000	35126.000	32640.000	34930.000
40	.000	32640.000	34930.000	35481.000
41	.000	34930.000	35481.000	38334.000
42	.000	35481.000	38334.000	40450.000
43	.000	38334.000	40450.000	38177.000
44	.000	40450.000	38177.000	37945.000
45	.000	38177.000	37945.000	36130.000
46	.000	37945.000	36130.000	36153.000
47	.000	36130.000	36153.000	36035.000
48	.000	36153.000	36035.000	31915.000
49	.000	36035.000	31915.000	33801.000
50	.000	31915.000	33801.000	32959.000
51	.000	33801.000	32959.000	35258.000
52	.000	32959.000	35258.000	34402.000
53	.000	35258.000	34402.000	37973.000
54	.000	34402.000	37973.000	39225.000
55	.000	37973.000	39225.000	38177.000
56	.000	39225.000	38177.000	37539.000
57	.000	38177.000	37539.000	35250.000
58	.000	37539.000	35250.000	37046.000
59	.000	35250.000	37046.000	36093.000
60	.000	37046.000	36093.000	32112.000
61	.000	36093.000	32112.000	34481.000
62	.000	32112.000	34481.000	33584.000

©2019 Gemmill A et al. *JAMA Network Open*

```
    63         .000 34481.000 33584.000 35477.000
    64         .000 33584.000 35477.000 35239.000
    65         .000 35477.000 35239.000 38802.000
    66         .000 35239.000 38802.000 39257.000
    67         .000 38802.000 39257.000 39456.000
    68         .000 39257.000 39456.000 38102.000
    69         .000 39456.000 38102.000 35635.000
    70         .000 38102.000 35635.000 37664.000
    71         .000 35635.000 37664.000 36289.000
    72         .000 37664.000 36289.000 33019.000
    73         .000 36289.000 33019.000 35657.000
    74         .000 33019.000 35657.000 34017.000
    75         .000 35657.000 34017.000 35373.000
    76         .000 34017.000 35373.000 35881.000
    77         .000 35373.000 35881.000 38919.000
    78         .000 35881.000 38919.000 39782.000
    79         .000 38919.000 39782.000 39664.000
    80         .000 39782.000 39664.000 37838.000
    81         .000 39664.000 37838.000 35923.000
    82         .000 37838.000 35923.000 37928.000
    83         .000 35923.000 37928.000 35950.000
    84         .000 37928.000 35950.000 33817.000
    85         .000 35950.000 33817.000 35373.000
    86         .000 33817.000 35373.000 33827.000
    87         .000 35373.000 33827.000 35347.000
    88         .000 33827.000 35347.000 35940.000
    89         .000 35347.000 35940.000 37820.000
    90         .000 35940.000 37820.000 40412.000
    91         .000 37820.000 40412.000 39212.000
    92         .000 40412.000 39212.000 37375.000
    93         .000 39212.000 37375.000 35906.000
    94         .000 37375.000 35906.000 36808.000
    95        1.000 35906.000 36808.000 35472.000
    96         .000 36808.000 35472.000 32066.000
    97         .000 35472.000 32066.000 34827.000
    98         .000 32066.000 34827.000 32407.000
    99         .000 34827.000 32407.000 35036.000
   100         .000 32407.000 35036.000 35258.000
   101         .000 35036.000 35258.000 37494.000
   102         .000 35258.000 37494.000 39344.000
   103         .000 37494.000 39344.000 38248.000
--

des dsmalls



VARIABLE    NAME    IS   DSMALLS
NUMBER OF OBSERVATIONS       103
NUMBER OF MISSING VALUES       0

                   STATISTIC      STD. ERROR     STATISTIC/S.E.

MEAN            163357.7184        802.7747        203.4914
VARIANCE      66378069.8709
STD DEVIATION     8147.2738
C.  V.              .0499
SKEWNESS           -.1709           .2379
KURTOSIS           -.2658           .4716

                   QUARTILE
```

©2019 Gemmill A et al. *JAMA Network Open*

```
MINIMUM         142759.0000
1ST QUARTILE    158385.0000
MEDIAN          162565.0000
3RD QUARTILE    169610.0000
MAXIMUM         181728.0000

                     RANGE

MAX - MIN        38969.0000
Q3  - Q1         11225.0000


--


des dsfalls



VARIABLE    NAME    IS   DSFALLS
NUMBER OF OBSERVATIONS       103
NUMBER OF MISSING VALUES       0

                   STATISTIC      STD. ERROR     STATISTIC/S.E.

MEAN            155678.4660        771.4831        201.7911
VARIANCE      61304182.1140
STD DEVIATION     7829.6987
C.  V.               .0503
SKEWNESS            -.1216           .2379
KURTOSIS            -.3106           .4716

                   QUARTILE

MINIMUM         136118.0000
1ST QUARTILE    150551.0000
MEDIAN          154723.0000
3RD QUARTILE    161889.0000
MAXIMUM         173357.0000

                     RANGE

MAX - MIN        37239.0000
Q3  - Q1         11338.0000


--


des dsmhisps



VARIABLE    NAME    IS  DSMHISPS
NUMBER OF OBSERVATIONS       103
NUMBER OF MISSING VALUES       0

                   STATISTIC      STD. ERROR     STATISTIC/S.E.

MEAN             38328.2621        239.2368        160.2105
VARIANCE       5895129.0385
STD DEVIATION     2427.9887
C.  V.               .0633
```

```
SKEWNESS              .1693           .2379
KURTOSIS             -.2243           .4716

                   QUARTILE

MINIMUM          32870.0000
1ST QUARTILE     36820.0000
MEDIAN           37849.0000
3RD QUARTILE     40064.0000
MAXIMUM          44597.0000

                      RANGE

MAX - MIN        11727.0000
Q3  - Q1          3244.0000


--


des dsfhisps



VARIABLE    NAME    IS  DSFHISPS
NUMBER OF OBSERVATIONS       103
NUMBER OF MISSING VALUES       0

                   STATISTIC      STD. ERROR     STATISTIC/S.E.

MEAN             36769.8252        234.5537        156.7651
VARIANCE       5666588.3417
STD DEVIATION     2380.4597
C.  V.              .0647
SKEWNESS            .1426           .2379
KURTOSIS           -.4045           .4716

                   QUARTILE

MINIMUM          31915.0000
1ST QUARTILE     35258.0000
MEDIAN           36690.0000
3RD QUARTILE     38392.7500
MAXIMUM          42821.0000

                      RANGE

MAX - MIN        10906.0000
Q3  - Q1          3134.7500


--


des nsmalls



VARIABLE    NAME    IS   NSMALLS
NUMBER OF OBSERVATIONS       103
NUMBER OF MISSING VALUES       0

                   STATISTIC      STD. ERROR     STATISTIC/S.E.
```

©2019 Gemmill A et al. *JAMA Network Open*

```
MEAN             16988.0291        103.9020        163.5005
VARIANCE       1111949.1070
STD DEVIATION     1054.4900
C.  V.               .0621
SKEWNESS             .2370           .2379
KURTOSIS           -.1511           .4716

                   QUARTILE

MINIMUM          14740.0000
1ST QUARTILE     16315.2500
MEDIAN           16953.0000
3RD QUARTILE     17737.7500
MAXIMUM          20089.0000

                      RANGE

MAX - MIN         5349.0000
Q3  - Q1          1422.5000


--


des nsfalls



VARIABLE    NAME    IS   NSFALLS
NUMBER OF OBSERVATIONS       103
NUMBER OF MISSING VALUES       0

                   STATISTIC      STD. ERROR     STATISTIC/S.E.

MEAN             14628.7282         92.1885        158.6828
VARIANCE        875368.2195
STD DEVIATION      935.6111
C.  V.               .0640
SKEWNESS             .3043           .2379
KURTOSIS             .0338           .4716

                   QUARTILE

MINIMUM          12667.0000
1ST QUARTILE     13930.0000
MEDIAN           14627.0000
3RD QUARTILE     15117.7500
MAXIMUM          17405.0000

                      RANGE

MAX - MIN         4738.0000
Q3  - Q1          1187.7500


--


des nsmhisps
```

©2019 Gemmill A et al. *JAMA Network Open*

```
VARIABLE    NAME    IS  NSMHISPS
NUMBER OF OBSERVATIONS       103
NUMBER OF MISSING VALUES       0

                   STATISTIC      STD. ERROR     STATISTIC/S.E.

MEAN              4226.4660         33.7070        125.3884
VARIANCE        117024.5454
STD DEVIATION      342.0885
C.  V.                .0809
SKEWNESS              .3906           .2379
KURTOSIS              .0652           .4716

                   QUARTILE

MINIMUM           3552.0000
1ST QUARTILE      3985.7500
MEDIAN            4220.0000
3RD QUARTILE      4394.2500
MAXIMUM           5253.0000

                      RANGE

MAX - MIN         1701.0000
Q3  - Q1           408.5000


--


des nsfhisps



VARIABLE    NAME    IS  NSFHISPS
NUMBER OF OBSERVATIONS       103
NUMBER OF MISSING VALUES       0

                   STATISTIC      STD. ERROR     STATISTIC/S.E.

MEAN              3548.4078         28.4964        124.5214
VARIANCE         83640.3811
STD DEVIATION      289.2065
C.  V.                .0815
SKEWNESS              .5568           .2379
KURTOSIS              .4099           .4716

                   QUARTILE

MINIMUM           3009.0000
1ST QUARTILE      3359.5000
MEDIAN            3524.0000
3RD QUARTILE      3685.7500
MAXIMUM           4467.0000

                      RANGE

MAX - MIN         1458.0000
Q3  - Q1           326.2500


--
```

©2019 Gemmill A et al. *JAMA Network Open*

```
nonhm=dsmalls-dsmhisps
--


nonhf=dsfalls-dsfhisps
--


nonhpm=nsmalls-nsmhisps
--


nonhpf=nsfalls-nsfhisps
--


des nonhm



VARIABLE    NAME    IS     NONHM
NUMBER OF OBSERVATIONS       103
NUMBER OF MISSING VALUES       0

                   STATISTIC      STD. ERROR     STATISTIC/S.E.

MEAN            125029.4563        586.1328        213.3125
VARIANCE      35385819.6231
STD DEVIATION     5948.5981
C.  V.               .0476
SKEWNESS            -.2687           .2379
KURTOSIS            -.2445           .4716

                   QUARTILE

MINIMUM         109508.0000
1ST QUARTILE    121593.0000
MEDIAN          125234.0000
3RD QUARTILE    129559.5000
MAXIMUM         137131.0000

                      RANGE

MAX - MIN        27623.0000
Q3  - Q1          7966.5000


--


des nonhf



VARIABLE    NAME    IS     NONHF
NUMBER OF OBSERVATIONS       103
NUMBER OF MISSING VALUES       0

                   STATISTIC      STD. ERROR     STATISTIC/S.E.

MEAN            118908.6408        559.2828        212.6091
VARIANCE      32218118.2913
```

```
STD DEVIATION     5676.1006
C.  V.                .0477
SKEWNESS             -.2146           .2379
KURTOSIS             -.2428           .4716

                   QUARTILE

MINIMUM         104052.0000
1ST QUARTILE    115313.0000
MEDIAN          118810.0000
3RD QUARTILE    122895.8000
MAXIMUM         130536.0000

                      RANGE

MAX - MIN        26484.0000
Q3  - Q1          7582.7500


--


des nonhpm



VARIABLE    NAME    IS    NONHPM
NUMBER OF OBSERVATIONS       103
NUMBER OF MISSING VALUES       0

                   STATISTIC      STD. ERROR     STATISTIC/S.E.

MEAN             12761.5631         71.4830        178.5259
VARIANCE        526311.1504
STD DEVIATION      725.4731
C.  V.                .0568
SKEWNESS              .1536           .2379
KURTOSIS             -.2558           .4716

                   QUARTILE

MINIMUM          11182.0000
1ST QUARTILE     12276.5000
MEDIAN           12733.0000
3RD QUARTILE     13242.5000
MAXIMUM          14836.0000

                      RANGE

MAX - MIN         3654.0000
Q3  - Q1           966.0000


--


des nonhpf



VARIABLE    NAME    IS    NONHPF
NUMBER OF OBSERVATIONS       103
NUMBER OF MISSING VALUES       0
```

©2019 Gemmill A et al. *JAMA Network Open*

```
                   STATISTIC      STD. ERROR     STATISTIC/S.E.

MEAN            11080.3204         64.8137        170.9565
VARIANCE       432683.6512
STD DEVIATION      657.7869
C.  V.               .0594
SKEWNESS             .1719           .2379
KURTOSIS            -.1035           .4716

                   QUARTILE

MINIMUM           9613.0000
1ST QUARTILE     10621.0000
MEDIAN           11099.0000
3RD QUARTILE     11463.0000
MAXIMUM          12938.0000

                      RANGE

MAX - MIN         3325.0000
Q3  - Q1           842.0000


--


uts nam moda. Mod psmhis(12)=con+(0)psmnh(12)+@
(0)tsmhis(12)+(0)tsmhis1(12)+(0)tsmhis2(12)+noise

--


ues mod moda. Hol resi(modar). span 1,94


THE FOLLOWING ANALYSIS IS BASED ON TIME SPAN   1  THRU   94

NONLINEAR ESTIMATION TERMINATED DUE TO:
RELATIVE CHANGE IN (OBJECTIVE FUNCTION)**0.5 LESS THAN  .1000D-02


SUMMARY FOR UNIVARIATE TIME SERIES MODEL --   MODA


-----------------------------------------------------------------------
VARIABLE   TYPE OF    ORIGINAL     DIFFERENCING
          VARIABLE   OR CENTERED
                                       12
 PSMHIS    RANDOM     ORIGINAL     (1-B  )
                                       12
 PSMNH     RANDOM     ORIGINAL     (1-B  )
                                       12
 TSMHIS    RANDOM     ORIGINAL     (1-B  )
                                       12
TSMHIS1    RANDOM     ORIGINAL     (1-B  )
                                       12
TSMHIS2    RANDOM     ORIGINAL     (1-B  )
-----------------------------------------------------------------------


 PARAMETER   VARIABLE  NUM./  FACTOR  ORDER   CONS-     VALUE      STD      T
   LABEL       NAME    DENOM.                 TRAINT               ERROR  VALUE
```

```
  1   CON              CNST      1      0     NONE   -27.7007   14.3789  -1.93
  2           PSMNH    NUM.      1      0     NONE      .1301     .0549   2.37
  3           TSMHIS   NUM.      1      0     NONE      .0672     .0209   3.21
  4          TSMHIS1   NUM.      1      0     NONE      .0062     .0173    .36
  5          TSMHIS2   NUM.      1      0     NONE      .0237     .0161   1.47


EFFECTIVE NUMBER OF OBSERVATIONS . .            82
R-SQUARE . . . . . . . . . . . . . . .        .899
RESIDUAL STANDARD ERROR. . . . . . .   .110106E+03
--


uid modar

NAME OF THE SERIES . . . . . . . . . .         MODAR
TIME PERIOD ANALYZED . . . . . . . . . 13  TO    94
MEAN OF THE (DIFFERENCED) SERIES . . .         .0007
STANDARD DEVIATION OF THE SERIES . . .      110.1056
T-VALUE OF MEAN (AGAINST ZERO) . . . .         .0001


AUTOCORRELATIONS

  1- 12     .31  .36  .04  .01  .04  .12  .20  .12  .03 -.23 -.23 -.36
  ST.E.     .11  .12  .13  .13  .13  .13  .13  .14  .14  .14  .14  .15

 13- 24    -.06 -.07  .01 -.03 -.05 -.10  .01 -.01  .13  .01  .11 -.12
  ST.E.     .16  .16  .16  .16  .16  .16  .16  .16  .16  .16  .16  .16


PARTIAL AUTOCORRELATIONS

  1- 12     .31  .29 -.16 -.08  .12  .14  .12 -.05 -.11 -.28 -.10 -.17
  ST.E.     .11  .11  .11  .11  .11  .11  .11  .11  .11  .11  .11  .11

 13- 24     .15  .04 -.07 -.00  .11  .05  .17 -.05 -.00 -.24  .05 -.24
  ST.E.     .11  .11  .11  .11  .11  .11  .11  .11  .11  .11  .11  .11
--


uts nam mod1. Mod psmhis(12)=con+(0)psmnh(12)+@
(0)tsmhis(12)+(0)tsmhis1(12)+(0)tsmhis2(12)+(12)/(1)noise

--


ues mod mod1. Hol resi(mod1r). span 1,94


THE FOLLOWING ANALYSIS IS BASED ON TIME SPAN   1  THRU   94

NONLINEAR ESTIMATION TERMINATED DUE TO:
RELATIVE CHANGE IN (OBJECTIVE FUNCTION)**0.5 LESS THAN  .1000D-02


SUMMARY FOR UNIVARIATE TIME SERIES MODEL --   MOD1


-----------------------------------------------------------------------
VARIABLE   TYPE OF    ORIGINAL     DIFFERENCING
          VARIABLE   OR CENTERED
```

©2019 Gemmill A et al. *JAMA Network Open*

```
                                     12
 PSMHIS    RANDOM     ORIGINAL     (1-B  )
                                     12
 PSMNH     RANDOM     ORIGINAL     (1-B  )
                                     12
 TSMHIS    RANDOM     ORIGINAL     (1-B  )
                                     12
TSMHIS1    RANDOM     ORIGINAL     (1-B  )
                                     12
TSMHIS2    RANDOM     ORIGINAL     (1-B  )
-----------------------------------------------------------------------


 PARAMETER   VARIABLE  NUM./  FACTOR  ORDER   CONS-     VALUE      STD     T
   LABEL       NAME    DENOM.                 TRAINT              ERROR  VALUE

  1   CON              CNST      1      0     NONE   -34.1430    8.3632  -4.08
  2          PSMNH     NUM.      1      0     NONE      .1369     .0483   2.83
  3          TSMHIS    NUM.      1      0     NONE      .0696     .0172   4.04
  4         TSMHIS1    NUM.      1      0     NONE      .0069     .0127    .55
  5         TSMHIS2    NUM.      1      0     NONE      .0197     .0133   1.48
  6          PSMHIS     MA       1     12     NONE      .7670     .0871   8.80
  7          PSMHIS    D-AR      1      1     NONE      .2359     .1103   2.14


EFFECTIVE NUMBER OF OBSERVATIONS . .            81
R-SQUARE . . . . . . . . . . . . . .          .935
RESIDUAL STANDARD ERROR. . . . . . .   .881706E+02
--


uid mod1r

NAME OF THE SERIES . . . . . . . . . .        MOD1R
TIME PERIOD ANALYZED . . . . . . . . . 14  TO    94
MEAN OF THE (DIFFERENCED) SERIES . . .      -1.2807
STANDARD DEVIATION OF THE SERIES . . .      88.1613
T-VALUE OF MEAN (AGAINST ZERO) . . . .       -.1307


AUTOCORRELATIONS

  1- 12    -.07  .22 -.02  .01  .09  .02  .20  .03  .05 -.18 -.13  .05
  ST.E.     .11  .11  .12  .12  .12  .12  .12  .12  .12  .12  .13  .13

 13- 24    -.06 -.02 -.00 -.03 -.02 -.14  .06 -.07  .06 -.08  .06 -.12
  ST.E.     .13  .13  .13  .13  .13  .13  .13  .13  .13  .13  .13  .13


PARTIAL AUTOCORRELATIONS

  1- 12    -.07  .22  .01 -.04  .09  .04  .18  .05 -.02 -.21 -.19  .09
  ST.E.     .11  .11  .11  .11  .11  .11  .11  .11  .11  .11  .11  .11

 13- 24    -.00 -.12  .01  .04  .06 -.07  .06 -.04 -.02 -.05  .07 -.17
  ST.E.     .11  .11  .11  .11  .11  .11  .11  .11  .11  .11  .11  .11
--


filt old psmhis. New mod1f. mod mod1.


THE FOLLOWING ANALYSIS IS BASED ON TIME SPAN   1  THRU  103
```

©2019 Gemmill A et al. *JAMA Network Open*

```
SERIES  PSMHIS  IS FILTERED USING MODEL   MOD1  , THE RESULT IS IN MOD1F
--


PSMHEXP=psmhis-mod1f
--


input mexp,mobs,mmod1f.

MEXP    , A    9  BY   1 VARIABLE, IS STORED IN THE WORKSPACE
MOBS    , A    9  BY   1 VARIABLE, IS STORED IN THE WORKSPACE
MMOD1F  , A    9  BY   1 VARIABLE, IS STORED IN THE WORKSPACE
--


Des mexp



VARIABLE    NAME    IS      MEXP
NUMBER OF OBSERVATIONS         9
NUMBER OF MISSING VALUES       0

                   STATISTIC      STD. ERROR     STATISTIC/S.E.

MEAN              3944.0755         89.2193         44.2065
VARIANCE         71640.8103
STD DEVIATION      267.6580
C.  V.               .0679
SKEWNESS            -.6802           .7171
KURTOSIS            -.9502          1.3997

                   QUARTILE

MINIMUM           3425.7670
1ST QUARTILE      3691.2710
MEDIAN            3997.0270
3RD QUARTILE      4090.0140
MAXIMUM           4251.3840

                      RANGE

MAX - MIN          825.6167
Q3  - Q1           398.7434


--


Des mobs



VARIABLE    NAME    IS      MOBS
NUMBER OF OBSERVATIONS         9
NUMBER OF MISSING VALUES       0

                   STATISTIC      STD. ERROR     STATISTIC/S.E.

MEAN              4092.0000         84.2036         48.5965
VARIANCE         63812.2500
STD DEVIATION      252.6109
```

©2019 Gemmill A et al. *JAMA Network Open*

```
C.  V.                .0617
SKEWNESS             -.0396          .7171
KURTOSIS            -1.3091         1.3997

                   QUARTILE

MINIMUM            3698.0000
1ST QUARTILE       3838.2500
MEDIAN             4105.0000
3RD QUARTILE       4224.2500
MAXIMUM            4501.0000

                      RANGE

MAX - MIN           803.0000
Q3  - Q1            386.0000


--


Des mmod1f



VARIABLE    NAME    IS    MMOD1F
NUMBER OF OBSERVATIONS         9
NUMBER OF MISSING VALUES       0

                   STATISTIC      STD. ERROR     STATISTIC/S.E.

MEAN                147.9246         31.6400          4.6752
VARIANCE           9009.7784
STD DEVIATION        94.9199
C.  V.                .6417
SKEWNESS             -.2180          .7171
KURTOSIS            -1.4309         1.3997

                   QUARTILE

MINIMUM            -13.0740
1ST QUARTILE        67.2747
MEDIAN             145.0880
3RD QUARTILE       215.6662
MAXIMUM            272.2330

                      RANGE

MAX - MIN           285.3070
Q3  - Q1            148.3915


--


uts nam mod1a. Mod mod1f=(0)n16step(bin)+noise

--


ues mod mod1a. Hol resi(mod1ar).
```

©2019 Gemmill A et al. *JAMA Network Open*

```
** THE TIME SERIES TO BE ANALYZED HAVE MISSING DATA AT THE
** BEGINNING OR THE USER HAS SPECIFIED TIME SPAN, SOME
** GENERATED SERIES SUCH AS RESIDUALS, FITTED VALUES,
** DISTURBANCE, AND FILTERED SERIES WILL BE SHORTER THAN THE
** ORIGINAL SERIES.


THE FOLLOWING ANALYSIS IS BASED ON TIME SPAN  14  THRU  103

NONLINEAR ESTIMATION TERMINATED DUE TO:
RELATIVE CHANGE IN (OBJECTIVE FUNCTION)**0.5 LESS THAN  .1000D-02


SUMMARY FOR UNIVARIATE TIME SERIES MODEL --  MOD1A


-----------------------------------------------------------------------
VARIABLE   TYPE OF    ORIGINAL     DIFFERENCING
          VARIABLE   OR CENTERED

 MOD1F     RANDOM     ORIGINAL     NONE

N16STEP    BINARY     ORIGINAL     NONE
-----------------------------------------------------------------------


 PARAMETER   VARIABLE  NUM./  FACTOR  ORDER   CONS-     VALUE      STD     T
   LABEL       NAME    DENOM.                 TRAINT               ERROR  VALUE

  1          N16STEP   NUM.      1      0     NONE   147.8617   29.4323   5.02


EFFECTIVE NUMBER OF OBSERVATIONS . .            90
R-SQUARE . . . . . . . . . . . . . .          .204
RESIDUAL STANDARD ERROR. . . . . . .   .882969E+02
--


uid mod1ar

NAME OF THE SERIES . . . . . . . . . .        MOD1AR
TIME PERIOD ANALYZED . . . . . . . . .  1  TO    90
MEAN OF THE (DIFFERENCED) SERIES . . .      -1.1525
STANDARD DEVIATION OF THE SERIES . . .      88.2894
T-VALUE OF MEAN (AGAINST ZERO) . . . .       -.1238


AUTOCORRELATIONS

  1- 12    -.12  .21 -.03 -.05  .11 -.00  .22  .00  .06 -.17 -.14  .11
  ST.E.     .11  .11  .11  .11  .11  .11  .11  .12  .12  .12  .12  .12

 13- 24    -.10  .04 -.01 -.02 -.01 -.13  .05 -.07  .06 -.11  .08 -.12
  ST.E.     .12  .12  .12  .12  .12  .12  .13  .13  .13  .13  .13  .13


PARTIAL AUTOCORRELATIONS

  1- 12    -.12  .20  .01 -.10  .11  .05  .19  .04 -.00 -.19 -.19  .13
  ST.E.     .11  .11  .11  .11  .11  .11  .11  .11  .11  .11  .11  .11

 13- 24    -.03 -.11  .01  .04  .05 -.06  .02 -.04 -.01 -.09  .09 -.14
  ST.E.     .11  .11  .11  .11  .11  .11  .11  .11  .11  .11  .11  .11
```

©2019 Gemmill A et al. *JAMA Network Open*

```
--

oes mod mod1a

THE FOLLOWING ANALYSIS IS BASED ON TIME SPAN  14  THRU  103


SUMMARY FOR UNIVARIATE TIME SERIES MODEL --  MOD1A

-----------------------------------------------------------------------
VARIABLE   TYPE OF    ORIGINAL     DIFFERENCING
          VARIABLE   OR CENTERED

 MOD1F     RANDOM     ORIGINAL     NONE

N16STEP    BINARY     ORIGINAL     NONE
-----------------------------------------------------------------------

 PARAMETER   VARIABLE  NUM./  FACTOR  ORDER   CONS-     VALUE      STD     T
   LABEL       NAME    DENOM.                 TRAINT              ERROR  VALUE

  1          N16STEP   NUM.      1      0     NONE   147.8632   28.1576   5.25


SUMMARY OF OUTLIER DETECTION AND ADJUSTMENT

-------------------------------------
 TIME    ESTIMATE   T-VALUE    TYPE
-------------------------------------
   41    258.067      3.06      AO
-------------------------------------

TOTAL NUMBER OF OBSERVATIONS. . . . . . . . . . . . . . .            90
EFFECTIVE NUMBER OF OBSERVATIONS. . . . . . . . . . . . .            90
RESIDUAL STANDARD ERROR (WITHOUT OUTLIER ADJUSTMENT). .   .882969E+02
RESIDUAL STANDARD ERROR (WITH OUTLIER ADJUSTMENT) . . .   .844727E+02
--


uts nam mod1b. Mod mod1f=(0 to 8)nov16(bin)+noise

--


ues mod mod1b. Hol resi(mod1br).


** THE TIME SERIES TO BE ANALYZED HAVE MISSING DATA AT THE
** BEGINNING OR THE USER HAS SPECIFIED TIME SPAN, SOME
** GENERATED SERIES SUCH AS RESIDUALS, FITTED VALUES,
** DISTURBANCE, AND FILTERED SERIES WILL BE SHORTER THAN THE
** ORIGINAL SERIES.


THE FOLLOWING ANALYSIS IS BASED ON TIME SPAN  14  THRU  103

NONLINEAR ESTIMATION TERMINATED DUE TO:
RELATIVE CHANGE IN (OBJECTIVE FUNCTION)**0.5 LESS THAN  .1000D-02


SUMMARY FOR UNIVARIATE TIME SERIES MODEL --  MOD1B
```

©2019 Gemmill A et al. *JAMA Network Open*

```
----------------------------------------------------------------------
VARIABLE   TYPE OF    ORIGINAL     DIFFERENCING
           VARIABLE   OR CENTERED

 MOD1F     RANDOM     ORIGINAL     NONE

 NOV16     BINARY     ORIGINAL     NONE
----------------------------------------------------------------------


 PARAMETER   VARIABLE  NUM./  FACTOR  ORDER   CONS-     VALUE      STD     T
   LABEL       NAME    DENOM.                 TRAINT               ERROR  VALUE

  1           NOV16    NUM.      1      0     NONE    67.0222   82.5787    .81
  2           NOV16    NUM.      1      1     NONE   144.9590   82.5787   1.76
  3           NOV16    NUM.      1      2     NONE    68.0029   82.5787    .82
  4           NOV16    NUM.      1      3     NONE   272.1471   82.5787   3.30
  5           NOV16    NUM.      1      4     NONE   139.1369   82.5787   1.68
  6           NOV16    NUM.      1      5     NONE   173.4951   82.5787   2.10
  7           NOV16    NUM.      1      6     NONE   229.6301   82.5787   2.78
  8           NOV16    NUM.      1      7     NONE   -13.0084   82.5787   -.16
  9           NOV16    NUM.      1      8     NONE   249.3706   82.5787   3.02


EFFECTIVE NUMBER OF OBSERVATIONS . .            82
R-SQUARE . . . . . . . . . . . . . . .        .304
RESIDUAL STANDARD ERROR. . . . . . .   .825788E+02
--


uid mod1br

NAME OF THE SERIES . . . . . . . . . .        MOD1BR
TIME PERIOD ANALYZED . . . . . . . . .  9  TO    90
MEAN OF THE (DIFFERENCED) SERIES . . .         .4969
STANDARD DEVIATION OF THE SERIES . . .       82.5773
T-VALUE OF MEAN (AGAINST ZERO) . . . .         .0545


AUTOCORRELATIONS

  1- 12    -.00  .17  .04 -.01  .13  .00  .24  .03  .03 -.16 -.15  .10
  ST.E.     .11  .11  .11  .11  .11  .12  .12  .12  .12  .12  .12  .13

 13- 24    -.14  .03 -.04 -.00 -.02 -.18  .11 -.11  .02  .02 -.01 -.04
  ST.E.     .13  .13  .13  .13  .13  .13  .13  .13  .13  .13  .13  .13


PARTIAL AUTOCORRELATIONS

  1- 12    -.00  .17  .05 -.04  .12  .01  .21  .02 -.04 -.21 -.17  .11
  ST.E.     .11  .11  .11  .11  .11  .11  .11  .11  .11  .11  .11  .11

 13- 24    -.09 -.05  .00  .06  .07 -.10  .08 -.07 -.05  .06  .01 -.14
  ST.E.     .11  .11  .11  .11  .11  .11  .11  .11  .11  .11  .11  .11
--


uts nam modb. Mod psfhis(12)=con+(0)psfnh(12)+@
(0)tsfhis(12)+(0)tsfhis1(12)+(0)tsfhis2(12)+noise

--
```

```
ues mod modb. Hol resi(modbr). span 1,94


THE FOLLOWING ANALYSIS IS BASED ON TIME SPAN   1  THRU   94

NONLINEAR ESTIMATION TERMINATED DUE TO:
RELATIVE CHANGE IN (OBJECTIVE FUNCTION)**0.5 LESS THAN  .1000D-02


SUMMARY FOR UNIVARIATE TIME SERIES MODEL --   MODB


-----------------------------------------------------------------------
VARIABLE   TYPE OF    ORIGINAL     DIFFERENCING
          VARIABLE   OR CENTERED
                                       12
 PSFHIS    RANDOM     ORIGINAL     (1-B  )
                                       12
 PSFNH     RANDOM     ORIGINAL     (1-B  )
                                       12
 TSFHIS    RANDOM     ORIGINAL     (1-B  )
                                       12
TSFHIS1    RANDOM     ORIGINAL     (1-B  )
                                       12
TSFHIS2    RANDOM     ORIGINAL     (1-B  )
-----------------------------------------------------------------------


 PARAMETER   VARIABLE  NUM./  FACTOR  ORDER   CONS-     VALUE      STD     T
   LABEL       NAME    DENOM.                 TRAINT               ERROR  VALUE

  1   CON              CNST      1      0     NONE   -24.2229   11.4199  -2.12
  2          PSFNH     NUM.      1      0     NONE      .1087     .0479   2.27
  3          TSFHIS    NUM.      1      0     NONE      .0768     .0177   4.34
  4         TSFHIS1    NUM.      1      0     NONE      .0151     .0139   1.08
  5         TSFHIS2    NUM.      1      0     NONE      .0057     .0135    .42


EFFECTIVE NUMBER OF OBSERVATIONS . .            82
R-SQUARE . . . . . . . . . . . . . .          .908
RESIDUAL STANDARD ERROR. . . . . . .   .888259E+02
--


uid modbr

NAME OF THE SERIES . . . . . . . . . .        MODBR
TIME PERIOD ANALYZED . . . . . . . . . 13  TO    94
MEAN OF THE (DIFFERENCED) SERIES . . .       -.0002
STANDARD DEVIATION OF THE SERIES . . .      88.8259
T-VALUE OF MEAN (AGAINST ZERO) . . . .        .0000


AUTOCORRELATIONS

  1- 12      .08 -.01  .13  .11  .01  .04  .07  .01  .01  .26  .06 -.33
  ST.E.      .11  .11  .11  .11  .11  .11  .11  .12  .12  .12  .12  .12

 13- 24      .02  .04 -.12 -.07  .13 -.17 -.06  .01  .03 -.14 -.03 -.03
  ST.E.      .13  .13  .13  .13  .13  .14  .14  .14  .14  .14  .14  .14
```

©2019 Gemmill A et al. *JAMA Network Open*

```
PARTIAL AUTOCORRELATIONS

  1- 12     .08 -.02  .13  .09  .00  .03  .05 -.01  .00  .24  .01 -.35
  ST.E.     .11  .11  .11  .11  .11  .11  .11  .11  .11  .11  .11  .11

 13- 24     .01 -.00 -.07 -.02  .15 -.22  .01 -.03  .04  .05  .04 -.22
  ST.E.     .11  .11  .11  .11  .11  .11  .11  .11  .11  .11  .11  .11
--


uts nam mod2. Mod psfhis(12)=con+(0)psfnh(12)+@
(0)tsfhis(12)+(0)tsfhis1(12)+(0)tsfhis2(12)+(10)(12)noise

--


ues mod mod2. Hol resi(mod2r). span 1,94


THE FOLLOWING ANALYSIS IS BASED ON TIME SPAN   1  THRU   94

NONLINEAR ESTIMATION TERMINATED DUE TO:
RELATIVE CHANGE IN (OBJECTIVE FUNCTION)**0.5 LESS THAN  .1000D-02


SUMMARY FOR UNIVARIATE TIME SERIES MODEL --   MOD2


-----------------------------------------------------------------------
VARIABLE   TYPE OF    ORIGINAL     DIFFERENCING
          VARIABLE   OR CENTERED
                                       12
 PSFHIS    RANDOM     ORIGINAL     (1-B  )
                                       12
 PSFNH     RANDOM     ORIGINAL     (1-B  )
                                       12
 TSFHIS    RANDOM     ORIGINAL     (1-B  )
                                       12
TSFHIS1    RANDOM     ORIGINAL     (1-B  )
                                       12
TSFHIS2    RANDOM     ORIGINAL     (1-B  )
-----------------------------------------------------------------------


 PARAMETER   VARIABLE  NUM./  FACTOR  ORDER   CONS-     VALUE      STD     T
   LABEL       NAME    DENOM.                 TRAINT               ERROR  VALUE

  1   CON              CNST      1      0     NONE   -22.8078    7.1604  -3.19
  2          PSFNH     NUM.      1      0     NONE      .1463     .0468   3.13
  3          TSFHIS    NUM.      1      0     NONE      .0676     .0149   4.55
  4         TSFHIS1    NUM.      1      0     NONE      .0195     .0089   2.21
  5         TSFHIS2    NUM.      1      0     NONE      .0067     .0092    .72
  6          PSFHIS     MA       1     10     NONE     -.3915     .1116  -3.51
  7          PSFHIS     MA       2     12     NONE      .6560     .0956   6.86


EFFECTIVE NUMBER OF OBSERVATIONS . .            82
R-SQUARE . . . . . . . . . . . . . .          .936
RESIDUAL STANDARD ERROR. . . . . . .   .742454E+02
--
```

```
uid mod2r

NAME OF THE SERIES . . . . . . . . . .         MOD2R
TIME PERIOD ANALYZED . . . . . . . . . 13  TO    94
MEAN OF THE (DIFFERENCED) SERIES . . .        .1956
STANDARD DEVIATION OF THE SERIES . . .      74.2451
T-VALUE OF MEAN (AGAINST ZERO) . . . .        .0239


AUTOCORRELATIONS

  1- 12     .15  .09  .10  .04  .04 -.07  .01  .01  .03 -.03  .00  .05
  ST.E.     .11  .11  .11  .11  .11  .11  .12  .12  .12  .12  .12  .12

 13- 24     .07  .02 -.08 -.10 -.01 -.16 -.07 -.06  .05  .01 -.01 -.09
  ST.E.     .12  .12  .12  .12  .12  .12  .12  .12  .12  .12  .12  .12


PARTIAL AUTOCORRELATIONS

  1- 12     .15  .06  .08  .01  .03 -.09  .02  .01  .04 -.04  .01  .04
  ST.E.     .11  .11  .11  .11  .11  .11  .11  .11  .11  .11  .11  .11

 13- 24     .07 -.01 -.09 -.10  .03 -.14  .01 -.04  .09 -.01  .02 -.14
  ST.E.     .11  .11  .11  .11  .11  .11  .11  .11  .11  .11  .11  .11
--


filt old psfhis. New mod2f. mod mod2.


THE FOLLOWING ANALYSIS IS BASED ON TIME SPAN   1  THRU  103
SERIES  PSFHIS  IS FILTERED USING MODEL   MOD2  , THE RESULT IS IN MOD2F
--


uts nam mod2b. Mod mod2f=(0)n16step(bin)+noise

--


ues mod mod2b. Hol resi(mod2br).


** THE TIME SERIES TO BE ANALYZED HAVE MISSING DATA AT THE
** BEGINNING OR THE USER HAS SPECIFIED TIME SPAN, SOME
** GENERATED SERIES SUCH AS RESIDUALS, FITTED VALUES,
** DISTURBANCE, AND FILTERED SERIES WILL BE SHORTER THAN THE
** ORIGINAL SERIES.


THE FOLLOWING ANALYSIS IS BASED ON TIME SPAN  13  THRU  103

NONLINEAR ESTIMATION TERMINATED DUE TO:
RELATIVE CHANGE IN (OBJECTIVE FUNCTION)**0.5 LESS THAN  .1000D-02


SUMMARY FOR UNIVARIATE TIME SERIES MODEL --  MOD2B


-----------------------------------------------------------------------
VARIABLE   TYPE OF    ORIGINAL     DIFFERENCING
          VARIABLE   OR CENTERED
```

©2019 Gemmill A et al. *JAMA Network Open*

```
 MOD2F     RANDOM     ORIGINAL     NONE

N16STEP    BINARY     ORIGINAL     NONE
-----------------------------------------------------------------------


 PARAMETER   VARIABLE  NUM./  FACTOR  ORDER   CONS-     VALUE      STD     T
   LABEL       NAME    DENOM.                 TRAINT               ERROR  VALUE

  1          N16STEP   NUM.      1      0     NONE   110.7763   23.9278   4.63


EFFECTIVE NUMBER OF OBSERVATIONS . .            91
R-SQUARE . . . . . . . . . . . . . .          .175
RESIDUAL STANDARD ERROR. . . . . . .   .717834E+02
--


uid mod2br

NAME OF THE SERIES . . . . . . . . . .        MOD2BR
TIME PERIOD ANALYZED . . . . . . . . .  1  TO    91
MEAN OF THE (DIFFERENCED) SERIES . . .         .1764
STANDARD DEVIATION OF THE SERIES . . .       71.7831
T-VALUE OF MEAN (AGAINST ZERO) . . . .         .0234


AUTOCORRELATIONS

  1- 12     .15  .08  .09  .04  .05 -.06 -.00  .01  .05 -.06 -.00  .04
  ST.E.     .10  .11  .11  .11  .11  .11  .11  .11  .11  .11  .11  .11

 13- 24     .07  .03 -.10 -.09  .03 -.16 -.09 -.09  .06  .03  .01 -.08
  ST.E.     .11  .11  .11  .11  .11  .11  .12  .12  .12  .12  .12  .12


PARTIAL AUTOCORRELATIONS

  1- 12     .15  .06  .07  .02  .03 -.09  .01  .01  .06 -.07  .02  .04
  ST.E.     .10  .10  .10  .10  .10  .10  .10  .10  .10  .10  .10  .10

 13- 24     .06  .00 -.10 -.09  .06 -.16 -.01 -.06  .11  .01  .04 -.13
  ST.E.     .10  .10  .10  .10  .10  .10  .10  .10  .10  .10  .10  .10
--


oes mod mod2b

THE FOLLOWING ANALYSIS IS BASED ON TIME SPAN  13  THRU  103


SUMMARY FOR UNIVARIATE TIME SERIES MODEL --  MOD2B

-----------------------------------------------------------------------
VARIABLE   TYPE OF    ORIGINAL     DIFFERENCING
          VARIABLE   OR CENTERED

 MOD2F     RANDOM     ORIGINAL     NONE

N16STEP    BINARY     ORIGINAL     NONE
-----------------------------------------------------------------------
```

©2019 Gemmill A et al. *JAMA Network Open*

```
 PARAMETER   VARIABLE  NUM./  FACTOR  ORDER   CONS-     VALUE      STD     T
   LABEL       NAME    DENOM.                 TRAINT              ERROR  VALUE

  1          N16STEP   NUM.      1      0     NONE   110.7774   22.9210   4.83


SUMMARY OF OUTLIER DETECTION AND ADJUSTMENT

-------------------------------------
 TIME    ESTIMATE   T-VALUE    TYPE
-------------------------------------
   63   -208.229     -3.03      AO
-------------------------------------

TOTAL NUMBER OF OBSERVATIONS. . . . . . . . . . . . . .            91
EFFECTIVE NUMBER OF OBSERVATIONS. . . . . . . . . . . .            91
RESIDUAL STANDARD ERROR (WITHOUT OUTLIER ADJUSTMENT). .   .717834E+02
RESIDUAL STANDARD ERROR (WITH OUTLIER ADJUSTMENT) . . .   .687629E+02
--


uts nam mod2c. Mod mod2f=(0 to 8)nov16(bin)+noise

--


ues mod mod2c. Hol resi(mod2cr).


** THE TIME SERIES TO BE ANALYZED HAVE MISSING DATA AT THE
** BEGINNING OR THE USER HAS SPECIFIED TIME SPAN, SOME
** GENERATED SERIES SUCH AS RESIDUALS, FITTED VALUES,
** DISTURBANCE, AND FILTERED SERIES WILL BE SHORTER THAN THE
** ORIGINAL SERIES.


THE FOLLOWING ANALYSIS IS BASED ON TIME SPAN  13  THRU  103

NONLINEAR ESTIMATION TERMINATED DUE TO:
RELATIVE CHANGE IN (OBJECTIVE FUNCTION)**0.5 LESS THAN  .1000D-02


SUMMARY FOR UNIVARIATE TIME SERIES MODEL --  MOD2C


-----------------------------------------------------------------------
VARIABLE   TYPE OF    ORIGINAL     DIFFERENCING
          VARIABLE   OR CENTERED

 MOD2F     RANDOM     ORIGINAL     NONE

 NOV16     BINARY     ORIGINAL     NONE
-----------------------------------------------------------------------


 PARAMETER   VARIABLE  NUM./  FACTOR  ORDER   CONS-     VALUE      STD     T
   LABEL       NAME    DENOM.                 TRAINT              ERROR  VALUE

  1           NOV16    NUM.      1      0     NONE   149.5927   69.5361   2.15
  2           NOV16    NUM.      1      1     NONE   115.4649   69.5361   1.66
  3           NOV16    NUM.      1      2     NONE   103.7139   69.5361   1.49
  4           NOV16    NUM.      1      3     NONE   178.4997   69.5361   2.57
  5           NOV16    NUM.      1      4     NONE   100.7980   69.5361   1.45
```

©2019 Gemmill A et al. *JAMA Network Open*

```
  6           NOV16    NUM.      1      5     NONE    69.4913   69.5361   1.00
  7           NOV16    NUM.      1      6     NONE    63.9759   69.5361    .92
  8           NOV16    NUM.      1      7     NONE    49.7765   69.5361    .72
  9           NOV16    NUM.      1      8     NONE   165.6736   69.5361   2.38


EFFECTIVE NUMBER OF OBSERVATIONS . .            83
R-SQUARE . . . . . . . . . . . . . .          .225
RESIDUAL STANDARD ERROR. . . . . . .   .695361E+02
--


uid mod2cr

NAME OF THE SERIES . . . . . . . . . .        MOD2CR
TIME PERIOD ANALYZED . . . . . . . . .  9  TO    91
MEAN OF THE (DIFFERENCED) SERIES . . .       1.6277
STANDARD DEVIATION OF THE SERIES . . .      69.5171
T-VALUE OF MEAN (AGAINST ZERO) . . . .        .2133


AUTOCORRELATIONS

  1- 12     .18  .13  .10  .04  .04 -.01  .07  .07  .01 -.04  .04 -.00
  ST.E.     .11  .11  .12  .12  .12  .12  .12  .12  .12  .12  .12  .12

 13- 24     .02 -.02 -.06 -.09 -.01 -.13 -.05 -.11  .08  .03 -.07 -.11
  ST.E.     .12  .12  .12  .12  .12  .12  .12  .12  .12  .12  .12  .12


PARTIAL AUTOCORRELATIONS

  1- 12     .18  .11  .06  .00  .02 -.03  .07  .05 -.02 -.07  .05 -.01
  ST.E.     .11  .11  .11  .11  .11  .11  .11  .11  .11  .11  .11  .11

 13- 24     .02 -.03 -.07 -.09  .04 -.11 -.01 -.09  .15  .02 -.06 -.13
  ST.E.     .11  .11  .11  .11  .11  .11  .11  .11  .11  .11  .11  .11
--


input stepf

STEPF   , A    9  BY   1 VARIABLE, IS STORED IN THE WORKSPACE
--


Des stepf



VARIABLE    NAME    IS     STEPF
NUMBER OF OBSERVATIONS        9
NUMBER OF MISSING VALUES      0

                   STATISTIC      STD. ERROR     STATISTIC/S.E.

MEAN             3429.7778         66.7622         51.3731
VARIANCE        40114.6944
STD DEVIATION     200.2865
C.  V.               .0584
SKEWNESS            -.5564           .7171
KURTOSIS           -1.2326          1.3997
```

©2019 Gemmill A et al. *JAMA Network Open*

```
                    QUARTILE

MINIMUM           3110.0000
1ST QUARTILE      3177.7500
MEDIAN            3497.0000
3RD QUARTILE      3536.7500
MAXIMUM           3691.0000

                       RANGE

MAX - MIN          581.0000
Q3  - Q1           359.0000


--


PSfHEXP=psfhis-mod2f
--


Prin years,months,psfhis,psfhexp,psmhis,psmhexp

YEARS    IS  A   103  BY     1  VARIABLE
MONTHS   IS  A   103  BY     1  VARIABLE
PSFHIS   IS  A   103  BY     1  VARIABLE
PSFHEXP  IS  A   103  BY     1  VARIABLE
PSMHIS   IS  A   103  BY     1  VARIABLE
PSMHEXP  IS  A   103  BY     1  VARIABLE
```

VARIABLE	YEARS	MONTHS	PSFHIS	PSFHEXP	PSMHIS	PSMHEXP
COLUMN-->	1	1	1	1	1	1
ROW						
1	2009.000	1.000	4186.000	***	4969.000	***
2	2009.000	2.000	3678.000	***	4274.000	***
3	2009.000	3.000	4179.000	***	4806.000	***
4	2009.000	4.000	4068.000	***	4722.000	***
5	2009.000	5.000	4228.000	***	4937.000	***
6	2009.000	6.000	4287.000	***	5062.000	***
7	2009.000	7.000	4467.000	***	5253.000	***
8	2009.000	8.000	3975.000	***	4948.000	***
9	2009.000	9.000	3720.000	***	4344.000	***
10	2009.000	10.000	4055.000	***	4821.000	***
11	2009.000	11.000	3542.000	***	4370.000	***
12	2009.000	12.000	3980.000	***	4677.000	***
13	2010.000	1.000	3863.000	3775.954	4625.000	***
14	2010.000	2.000	3360.000	3344.503	3995.000	3968.867
15	2010.000	3.000	3856.000	3945.289	4440.000	4501.207
16	2010.000	4.000	3625.000	3709.634	4274.000	4270.243
17	2010.000	5.000	3936.000	3861.382	4338.000	4498.478
18	2010.000	6.000	3841.000	3888.639	4692.000	4587.037
19	2010.000	7.000	3852.000	3981.820	4622.000	4777.671
20	2010.000	8.000	3805.000	3749.825	4614.000	4553.411
21	2010.000	9.000	3456.000	3478.450	4151.000	4113.557
22	2010.000	10.000	3956.000	3851.744	4681.000	4578.192
23	2010.000	11.000	3593.000	3540.557	4323.000	4333.215
24	2010.000	12.000	3878.000	3866.911	4508.000	4572.536
25	2011.000	1.000	3761.000	3614.911	4351.000	4445.580
26	2011.000	2.000	3254.000	3154.539	3811.000	3772.887
27	2011.000	3.000	3781.000	3664.367	4356.000	4254.964
28	2011.000	4.000	3296.000	3421.614	4068.000	4104.965
29	2011.000	5.000	3617.000	3692.407	4373.000	4317.970

©2019 Gemmill A et al. *JAMA Network Open*

30	2011.000	6.000	3760.000	3831.469	4633.000	4627.254
31	2011.000	7.000	3704.000	3779.675	4588.000	4588.333
32	2011.000	8.000	3706.000	3689.641	4348.000	4516.791
33	2011.000	9.000	3285.000	3260.171	3958.000	3860.737
34	2011.000	10.000	3729.000	3653.432	4345.000	4252.593
35	2011.000	11.000	3371.000	3370.717	4123.000	4114.581
36	2011.000	12.000	3600.000	3639.455	4209.000	4243.474
37	2012.000	1.000	3654.000	3593.593	4099.000	4226.446
38	2012.000	2.000	3141.000	3153.940	3798.000	3710.482
39	2012.000	3.000	3472.000	3478.684	4220.000	4119.113
40	2012.000	4.000	3250.000	3185.087	3787.000	3866.291
41	2012.000	5.000	3524.000	3588.437	4476.000	4217.933
42	2012.000	6.000	3516.000	3648.397	4385.000	4431.266
43	2012.000	7.000	3778.000	3776.460	4550.000	4519.015
44	2012.000	8.000	3684.000	3660.472	4404.000	4434.981
45	2012.000	9.000	3245.000	3194.393	3875.000	3789.449
46	2012.000	10.000	3674.000	3718.016	4475.000	4383.241
47	2012.000	11.000	3481.000	3371.531	4087.000	4036.809
48	2012.000	12.000	3586.000	3509.701	4314.000	4141.634
49	2013.000	1.000	3526.000	3540.924	4152.000	4210.311
50	2013.000	2.000	3024.000	2951.293	3558.000	3501.134
51	2013.000	3.000	3358.000	3351.253	3951.000	4040.606
52	2013.000	4.000	3170.000	3154.626	3634.000	3812.499
53	2013.000	5.000	3440.000	3546.953	4082.000	4188.097
54	2013.000	6.000	3491.000	3434.010	4107.000	4162.219
55	2013.000	7.000	3636.000	3695.303	4482.000	4444.095
56	2013.000	8.000	3569.000	3513.254	4240.000	4307.077
57	2013.000	9.000	3084.000	3161.216	3796.000	3662.323
58	2013.000	10.000	3497.000	3639.421	4225.000	4371.004
59	2013.000	11.000	3250.000	3255.233	3827.000	3982.699
60	2013.000	12.000	3501.000	3611.521	4107.000	4207.826
61	2014.000	1.000	3506.000	3553.932	4151.000	4174.656
62	2014.000	2.000	3009.000	3027.599	3564.000	3599.284
63	2014.000	3.000	3152.000	3360.229	3927.000	3984.967
64	2014.000	4.000	3212.000	3284.724	3771.000	3799.471
65	2014.000	5.000	3518.000	3487.543	4122.000	4193.638
66	2014.000	6.000	3498.000	3549.984	4124.000	4199.819
67	2014.000	7.000	3655.000	3686.178	4499.000	4450.437
68	2014.000	8.000	3398.000	3447.360	4103.000	4246.865
69	2014.000	9.000	3154.000	3217.358	3882.000	3771.780
70	2014.000	10.000	3556.000	3591.883	4333.000	4312.390
71	2014.000	11.000	3281.000	3265.765	3902.000	3930.290
72	2014.000	12.000	3497.000	3588.807	4289.000	4245.388
73	2015.000	1.000	3531.000	3405.962	4098.000	4154.488
74	2015.000	2.000	3065.000	2994.745	3552.000	3568.872
75	2015.000	3.000	3472.000	3397.076	4062.000	4006.456
76	2015.000	4.000	3160.000	3229.465	3870.000	3873.797
77	2015.000	5.000	3480.000	3445.474	4026.000	4101.369
78	2015.000	6.000	3483.000	3524.083	4214.000	4240.315
79	2015.000	7.000	3625.000	3697.502	4391.000	4477.546
80	2015.000	8.000	3457.000	3458.362	4250.000	4178.137
81	2015.000	9.000	3311.000	3180.346	3775.000	3890.065
82	2015.000	10.000	3588.000	3515.168	4252.000	4292.173
83	2015.000	11.000	3291.000	3358.152	3844.000	3868.320
84	2015.000	12.000	3581.000	3577.385	4309.000	4213.276
85	2016.000	1.000	3429.000	3485.734	4031.000	4083.055
86	2016.000	2.000	3224.000	3133.144	3735.000	3641.280
87	2016.000	3.000	3449.000	3393.347	4087.000	4035.618
88	2016.000	4.000	3183.000	3189.602	3818.000	3800.924
89	2016.000	5.000	3476.000	3425.537	4190.000	4067.452
90	2016.000	6.000	3593.000	3504.584	4250.000	4269.724
91	2016.000	7.000	3631.000	3588.139	4451.000	4332.513
92	2016.000	8.000	3645.000	3572.073	4381.000	4245.766

©2019 Gemmill A et al. *JAMA Network Open*

```
   93     2016.000     9.000  3118.000  3124.247  3844.000  3777.837
   94     2016.000    10.000  3589.000  3474.647  4161.000  4144.675
   95     2016.000    11.000  3363.000  3213.406  3914.000  3846.977
   96     2016.000    12.000  3581.000  3465.534  4238.000  4093.040
   97     2017.000     1.000  3500.000  3396.285  4065.000  3996.997
   98     2017.000     2.000  3116.000  2937.499  3698.000  3425.850
   99     2017.000     3.000  3461.000  3360.201  4105.000  3965.862
  100     2017.000     4.000  3110.000  3040.508  3813.000  3639.503
  101     2017.000     5.000  3497.000  3433.023  4311.000  4081.368
  102     2017.000     6.000  3549.000  3499.223  4183.000  4196.008
  103     2017.000     7.000  3691.000  3525.325  4501.000  4251.627
--

stop
```

©2019 Gemmill A et al. *JAMA Network Open*

eTable 1. Estimated Parameters for Step 1 Regressions and Step 2 Transfer Functions for Monthly Male and Female Preterm Births to Latina Women in the United States for the First 94 Months of the Obama Presidency

	Step 1 regressions		Step 2 transfer functions	
	Males	Females	Males	Females
Constant	-27.70 (14.38)	-24.22* (11.42)	-34.21** (8.39)	-22.81** (7.16)
Term births (differenced at) to Latina women at month t	0.07** (0.02)	0.08** (0.02)	0.07** (0.02)	0.07** (0.01)
Term births (differenced at 12) to Latina women at month t+1	0.01 (0.02)	0.01 (0.01)	0.01 (0.01)	0.02* (0.01)
Term Births (differenced at 12) to Latina women at month t+2	0.02 (0.0161)	0.01 (0.01)	0.02 (0.01)	0.01 (0.01)
Preterm births (differenced at 12) to other women at month t	0.13* (0.05)	0.11* (0.05)	0.14** (0.04)	0.15** (0.05)
Autoregression at p=1			0.23* (0.11)	none
Moving Average at q = 10<br>Moving Average at q = 12			none<br>0.77** (0.08)	-0.39** (0.11)<br>0.67** (0.09)

*p<.05, 2-tailed test

**p<.01, 2-tailed test

Standard errors are given in parentheses. Births were differenced at 12 months.

©2019 Gemmill A et al. *JAMA Network Open*

eTable 2. Estimated Parameters for Regressions of Male and Female Preterm Births to Latina Women on the Presidential Election of November 2016

	Male	Female
Constant	0.55 (9.66)	1.82 (8.08)
Election in month of births	66.46 (83.14)	147.76* (69.98)
1 month before births	144.40 (83.14)	113.64 (69.98)
2 months before births	67.44 (83.14)	101.88 (69.98)
3 months before births	271.58** (83.14)	176.67* (69.98)
4 months before births	138.57 (83.14)	98.97 (69.98)
5 months before births	172.93* (83.14)	67.66 (69.98)
6 months before births	229.07** (83.14)	62.15 (69.98)
7 months before births	-13.56 (83.14)	47.95 (69.98)
8 months before births	248.82** (83.14)	163.84* (69.98)

*$p<.05$, 2-tailed test

**$p<.01$, 2-tailed test

Regressions are adjusted for population at risk and preterm births to non-Latina women. Includes 103 months ending July 2017. Standard errors are given in parentheses.

©2019 Gemmill A et al. *JAMA Network Open*

eTable 3. Estimated Parameters for Full Transfer Functions for Monthly Male and Female Preterm Births to Latina women in the United States for the 103 Months Beginning January 2009

	Males	Females
Constant	-32.39** (8.25)	-23.27** (6.82)
Term births (differenced at 12 months) to Latina women at month t	0.06** (0.02)	0.14** (0.04)
Term births (differenced at 12 months) to Latina women at month t+1	0.01 (0.01)	0.02* (0.01)
Term Births (differenced at 12 months) to Latina women at month t+2	0.01 (0.01)	0.01 (0.01)
Preterm births (differenced at 12 months) to other women at month t	0.15** (0.05)	0.14* (0.04)
Election variable (scored 1 for November through July 2016 and 0 otherwise)	177.80** (41.73)	112.77** (27.82)
Autoregression at p=1	0.20* (0.10)	none
Moving Average at q = 10<br><br>Moving Average at q = 12	none<br><br>0.72** (0.09)	-0.36** (0.11)<br><br>0.66** (0.09)

*p<.05, 2-tailed test

**p<.01, 2-tailed test

Standard errors are given in parentheses. Functions are differenced at 12 months.

©2019 Gemmill A et al. *JAMA Network Open*

eReferences

1. Box G, Jenkins G, Reinsel G. *Time Series Analysis: Forecasting and Control*. 4th ed. Hoboken, NJ: Wiley, 2008.
2. Chang I, Tiao GC, Chen C. Estimation of time series parameters in the presence of outliers. *Technometrics* 1988;30:193-204.

©2019 Gemmill A et al. *JAMA Network Open*
